# Supplementary material for: Health-seeking behaviour and beliefs around sore throat in The Gambia: A qualitative study
Source: PLOS Glob Public Health. 2024 Mar 25;4(3):e0002257. doi: 10.1371/journal.pgph.0002257 (PMC10962789; doi:10.1371/journal.pgph.0002257)
Supplement: S1 Appendix — (DOCX) [file pgph.0002257.s002.docx]

## S1 Appendix: Interview Guideline

When the participant’s preferred language for the interview was any other than English, the nurse or fieldworker fluent in the preferred language would orally translate both questions and answers in English. The English translated parts were transcribed *verbatim*, with indications in the transcript document of when the participant was speaking in English. Alongside the transcript, notes regarding the participant’s interview summary, the positionality of the researchers and self-reflexivity thoughts were collected. All interviews were conducted in a quiet private area, elected by the participants, generally consisting of the compound’s outdoor communal area or the house’s living room, and lasted between 20 and 40 minutes. Transcripts were not returned to participants, however, some participants were re-visited and prompted for further clarifications after their interview audio was transcribed.

| **Version** | **3.0** | **Date** | **20^th^ July 2022** |
| --- | --- | --- | --- |

**Topic: Health-seeking behaviour, health literacy and beliefs around sore throats in The Gambia**

| SCC: |  |
| --- | --- |

Instructions:

- Follow the informed consent procedures
- If consent is given, audio record the interview
- This interview guide is to be used in a flexible manner
- The aim is to collect semi-structured information from the respondent
- The left-hand column lists the topic of interest
- The right-hand column contains a list of questions
- Use a flexible approach; use probes where needed
- It is not necessary to ask all these questions in the order listed; follow the responses/flow of the conversation
- Listen actively

| Topics | Possible questions and probes | |
| --- | --- | --- |
| Opening | Hello, my name is…   - Read out the information sheet - Obtained informed consent - Identify if the participant has any questions or concerns about the information on the consent form - Remind that the information will be considered confidential and how the information generated during the interview will be treated - Inform the participant that notes will be taken during the interview and that the interview will be recorded - Remind the participant, that he can interrupt and end the interview at any point or ask for a break | |
| Socio-demographic information | 1. Tell me about yourself  - What do you do to survive? - Prompt on: age, gender, number of children and age, other persons living in the household, languages spoken, literacy/education (number of school years), ethnic group, relationship to the children | |
| Illness perception  and interpretation | Previous experience & perceived susceptibility | 1. Tell me about your child’s experience with sore throat  - How did you first know that your child had sore throat? (How does the child behave, feel…?) - How long did the child have it for? - How did the illness stop? - How often does the child have it? |
|  | Knowledge of illness & perceived severity | 1. What causes the illness?  - Why does your child get the disease? - How do you treat sore throat? - Can the illness be dangerous for your child? (How & why?) |
| Decision making, treatment seeking and social values | TMG (therapy management group), social values, stigma, social pressure, social support | 1. What happened after your child started to have sore throat?  - What did you do? - How do you manage sore throat at home? Do you give any treatment?   - Which treatment, why [treatment], where do you get it from, how long do you administrate it for? - Did you seek for help?   - When, where, who and why? - Who accompanies the child if it needs to get health care? - What do other people say about sore throat? (who are those people) |
| Access to care and resource seeking  /  Barriers &  Facilitators | Availability and Accessibility | 1. How long does it take you to get from your home to the nearest    - Hospital    - Health center    - Pharmacy    - Traditional healer/informal sector    - Other  - Would you go to any of those facilities when your child has a sore throat? If YES: when, where, why and…   - Who would usually see you there? (doctor, nurse, pharmacist, traditional healer, other…)   - What kind of diagnostics and treatment do they usually have available for sore throat?   - How are these things helping your child to get better?   - Who pays for health care?   - How satisfied are you with the services provided there for sore throat?   - What happens after you leave the facility? |
| Concluding | Acute rheumatic fever | 1. Have you ever known someone with Acute Rheumatic Fever?  - What have you heard about Acute rheumatic fever? - How do you know if someone has acute rheumatic fever? |
|  | Rheumatic heart disease | 1. Have you ever known someone with Rheumatic Heart Disease?  - What have you heard about rheumatic heart disease? - How do you know if someone has rheumatic heart disease? |
| Closing | - Is there anything else you would like to say? - Do you have any questions? - Thank you for your time | |

**Participant’s Interview Summary**

Tape-record the in-depth study interviews and immediately after you have finished, fill in notes from the caregiver’s story from your memory on this form. If you have forgotten to ask anything then, you can go back to the patient and ask for more details from his or her story. Share notes with Study Coordinator and re-interview if necessary. Arrange to interview additional family members/neighbours/identified person of interest as necessary.

Interviewee ID: _____________ Interview conducted with: ________________

Interview date: _____________ Interviewer: ______________ Audio #: ______

Core elements of the caregiver treatment-seeking story: (when symptoms occurred; when/where/why treatments sought; who sleeked for treatment, who suggested treatments; treatment outcomes)

Core elements of the caregiver sore throat perception, beliefs and sensing: (what causes the sore throat, why does the child get it, perceived as dangerous?, what do other people say about it )

Availability and accessibility to health care facilities for sore throats ( identified barriers, use of the facility, relationship with the facility and its use):

Knowledge, beliefs and experience surrounding ARF:

Knowledge, beliefs and experience surrounding RHD:

Other information: (Eg. questions asked)

Behaviour of interviewee during interview:

Notes on interviewer behaviour during interview (Eg. things to improve):

Notes on other people to interview:
