# Supplementary material for: Health-seeking behaviour and beliefs around sore throat in The Gambia: A qualitative study
Source: PLOS Glob Public Health. 2024 Mar 25;4(3):e0002257. doi: 10.1371/journal.pgph.0002257 (PMC10962789; doi:10.1371/journal.pgph.0002257)
Supplement: S2 Appendix — (DOCX) [file pgph.0002257.s003.docx]

## S2 Appendix

### Participant information sheet for in-person-interview

| Version | 5.0 | Date | 18 July 2022 |
| --- | --- | --- | --- |

**Study Title:** Health-seeking behaviour and beliefs around sore throat in The Gambia: a qualitative study

| SCC: |  |
| --- | --- |

Sponsor: MRC Unit The Gambia at the London School of Hygiene and Tropical Medicine

**What is informed consent?**

You are invited to take part in a research study. The purpose of a research study is to gather information that may be useful in future for the whole population. Before you decide, you need to understand all the information about this study and what it will involve. Please take time to read the following information or get the information explained to you in your language. Listen carefully and feel free to ask if there is anything that you do not understand. Ask for it to be explained until you are satisfied. It is your choice to take part and you can stop at any time. You may also wish to consult your spouse, family members or others before deciding to take part in the study.

If you decide to take part in the study, you will need to sign or thumbprint a consent form saying you agree to be in the study. You will receive a copy of this.

**Why is this study being done?**

We would like to ask some questions to find out how much people know about sore throat, what they think about the risk of these conditions and their consequences in their children, and what parents usually do when their child suffers from one of these conditions in The Gambia. This information will help us to learn more about what people in The Gambia think about sore throat and health care for them.

**What does this study involve?**

If you agree to take part in the study and sign or thumbprint the consent form, we will ask you a series of questions and audio record your answers on a recording device (e.g. a phone). The interview procedure will include a part to check that you are eligible to take part, followed by some questions about your background, family, education and income. Then we will ask a series of questions about your understanding of sore throat, what you would normally do if a child of yours was sick and what your feelings about treatment and health care for them are.

The questions will be asked in Mandinka, English or your preferred local language. The answers that you give will be anonymous and kept confidential. The interview will be carried out in a quiet, private place, and should last about 30 minutes. If the time is not convenient for you or you would like to discuss the study with a spouse or your family first, we can arrange another time at your convenience to do the interview.

**What harm or discomfort can you expect in the study?**

You may find during the interview that some of the questions asked are challenging.

**Why am I being asked to take part in this study?**

You have been identified as an adult parent or caregiver of a child who has experienced a sore throat in the past month, as a key informant or as another identified community group of interest living in Sukuta (The Gambia) at the time of the study.

**What benefits can you expect in the study?**

There are no direct benefits to you for participating in this study, but the information gathered will help us to understand how sore throat are viewed in The Gambia, which may be useful in the future for planning public health campaigns to reduce the impact of streptococcus pyogenes. The results of the study will be made available to your community.

**Will you be compensated for participating in the study?**

You will not get paid for participation.

**What happens if you refuse to participate in the study or change your mind later?**

You are free to participate or not in the study and you will be free to stop the interview at any time without giving a reason.

**How will personal records remain confidential and who will have access to it?**

All information that is collected about you in the course of the study will be kept strictly confidential. Your personal information will only be available to the study team members and might be seen by some rightful persons from the Ethics Committee, Government authorities and sponsor. This could include organisations outside The Gambia. Anybody outside the study team who might see such information would only do so to ensure the study was being conducted properly.

Anonymous quotes could be used in future reports or publications. No personally identifiable information will be included in any data available via a repository.

**Who should you contact if you have questions?**

If you have any queries regarding the study you can contact Maria Suau Sans (396 62 81 OR 0034 646 56 77 78), and you can always call the personal numbers of the study staff given to you. If you have any concerns you can also contact staff at your health centre or clinic.

Please feel free to ask any question you might have about the research study.

**Who has reviewed this study?**

This study has been reviewed and approved by a panel of scientists at the Medical Research Council and the Gambia Government/MRC Joint Ethics Committee, which consists of scientists and lay persons to protect your rights and wellbeing. The Ethics Committee protects your rights and wellbeing and has given permission for it to take place.

### Participant information sheet for informal conversation

| Version | 5.0 | Date | 18 July 2022 |
| --- | --- | --- | --- |

Study Title: Health-seeking behaviour and beliefs around sore throat in The Gambia: a qualitative study

| SCC: |  |
| --- | --- |

Sponsor: MRC Unit The Gambia at the London School of Hygiene and Tropical Medicine

**What is informed consent?**

You are invited to take part in a research study. The purpose of a research study is to gather information that may be useful in future for the whole population. Before you decide, you need to understand all the information about this study and what it will involve. Please take time to read the following information or get the information explained to you in your language. Listen carefully and feel free to ask if there is anything that you do not understand. Ask for it to be explained until you are satisfied. It is your choice to take part and you can stop at any time. You may also wish to consult your spouse, family members or others before deciding to take part in the study.

If you decide to take part in the study, you will need to sign or thumbprint a consent form saying you agree to be in the study. You will receive a copy of this.

**Why is this study being done?**

We would like to ask some questions to find out how much people know about sore throat, which are and how to identify important community groups to ask about sore throat health care seeking behaviours and beliefs, what they think about the risk of these conditions and their consequences in their children, and what parents, caregivers and other community groups usually do when their child suffers from one of these conditions in The Gambia. This information will help us to learn more about what people in The Gambia think about sore throat and health care for them.

**What does this study involve?**

If you agree to take part in the study and sign or thumbprint the consent form, we will ask you a series of questions and take notes on your comments occasionally. The informal conversation procedure will start with a part to check that you are eligible to take part in the study. There is no structure to be followed in this conversation. We are interested in hearing about your background, your understanding of sore throat, what your feelings about treatment, health care for them are. This information might be used to later identify and contact relevant community groups or individuals to perform interviews about sore throat health care seeking behaviours and beliefs.

The questions will be asked in Mandinka, English or your preferred local language. The answers that you give will be anonymous and kept confidential. The informal conversation will be carried out in a quiet, private place, and should last about 30 minutes. If the time is not convenient for you or you would like to discuss the study with a spouse or your family first, we can arrange another time at your convenience to do the informal conversation.

**Why am I being asked to take part in this study?**

You have been identified as an adult parent or caregiver of a child who has experienced a sore throat in the past month, as a key informant or as another identified community group of interest living in Sukuta (The Gambia) at the time of the study.

**What harm or discomfort can you expect in the study?**

You may find during the informal conversation that some of the topics discussed are challenging.

**What benefits can you expect in the study?**

There are no direct benefits to you for participating in this study, but the information gathered will help us to understand how sore throat are viewed in The Gambia, which may be useful in the future for planning public health campaigns to reduce the impact of streptococcus pyogenes. The results of the study will be made available to your community.

**Will you be compensated for participating in the study?**

You will not get paid for participation.

**What happens if you refuse to participate in the study or change your mind later?**

You are free to participate or not in the study and you will be free to stop the informal conversation at any time without giving a reason.

**How will personal records remain confidential and who will have access to it?**

All information that is collected about you in the course of the study will be kept strictly confidential. Your personal information will only be available to the study team members and might be seen by some rightful persons from the Ethics Committee, Government authorities and sponsor. This could include organisations outside The Gambia. Anybody outside the study team who might see such information would only do so to ensure the study was being conducted properly.

Anonymous quotes could be used in future reports or publications. No personally identifiable information will be included in any data available via a repository.

**Who should you contact if you have questions?**

If you have any queries regarding the study you can contact Maria Suau Sans (396 62 81 OR 0034 646 56 77 78), and you can always call the personal numbers of the study staff given to you. If you have any concerns you can also contact staff at your health centre or clinic.

Please feel free to ask any question you might have about the research study.

**Who has reviewed this study?**

This study has been reviewed and approved by a panel of scientists at the Medical Research Council and the Gambia Government/MRC Joint Ethics Committee, which consists of scientists and lay persons to protect your rights and wellbeing. The Ethics Committee protects your rights and wellbeing and has given permission for it to take place.

### In-person-interview consent form

Participant Identification Number: |__|__|__| _____________________________

(Printed name of participant)

I have read the written information **OR**

I have had the information explained to me by study personnel in a language that I understand

and I:

confirm that my choice to participate is entirely voluntarily,

confirm that I have had the opportunity to ask questions about this study and I am satisfied with the answers and explanations that have been provided,

understand that I grant access to data about me to authorised persons described in the information sheet,

understand that I consent for anonymous quotes to be potentially used in reports / publications,

have received sufficient time to consider to take part in this study,

agree to take part in this study.

| Participant’s signature/ thumbprint* |  |  |  |  |  |
| --- | --- | --- | --- | --- | --- |
|  |  |  | Date (dd-mmm-yyyy) Time (24hr) | | |
|  |  |  |  | | |
| Printed name of person obtaining consent |  | | | | |
| **I attest that I have explained the study information accurately in ________________ to, and was understood to the best of my knowledge by, the participant and that he/she has freely given consent to participate.** | | | | | |
| Signature of person obtaining consent |  |  |  |  |  |
|  |  |  | Date (dd-mmm-yyyy) Time (24hr) | | |

** Only required if the participant is unable to read or write.*

A copy of this informed consent document has been provided to the participant.

## INFORMAL CONVERSATION CONSENT FORM

Participant Identification Number: |__|__|__| ______________________________

(Printed name of participant)

I have read the written information **OR**

I have had the information explained to me by study personnel in a language that I understand

and I:

confirm that my choice to participate is entirely voluntarily,

confirm that I have had the opportunity to ask questions about this study and I am satisfied with the answers and explanations that have been provided,

understand that I grant access to data about me to authorised persons described in the information sheet,

understand that I consent for anonymous quotes to be potentially used in reports / publications,

have received sufficient time to consider to take part in this study,

agree to take part in this study.

| Participant’s signature/ thumbprint* |  |  |  |  |  |
| --- | --- | --- | --- | --- | --- |
|  |  |  | Date (dd-mmm-yyyy) Time (24hr) | | |
|  |  |  |  | | |
| Printed name of person obtaining consent |  | | | | |
| **I attest that I have explained the study information accurately in ______________________ to, and was understood to the best of my knowledge by, the participant and that he/she has freely given consent to participate.** | | | | | |
| Signature of person obtaining consent |  |  |  |  |  |
|  |  |  | Date (dd-mmm-yyyy) Time (24hr) | | |

** Only required if the participant is unable to read or write.*

A copy of this informed consent document has been provided to the participant.
