# Supplementary material for: Health-seeking behaviour and beliefs around sore throat in The Gambia: A qualitative study
Source: PLOS Glob Public Health. 2024 Mar 25;4(3):e0002257. doi: 10.1371/journal.pgph.0002257 (PMC10962789; doi:10.1371/journal.pgph.0002257)
Supplement: S1 Table — (DOCX) [file pgph.0002257.s004.docx]

## **S1 Table**

COREQ checklist. This table was based on the criteria developed by Allison Tong, Peter Sainsbury and Jonathan Craig and previously used for qualitative studies by Hadija Nalubwama and David Watkins group.

| Item # | Guide questions/description | Response |
| --- | --- | --- |
| **Domain 1: Research team and reflexivity** | | |
| 1. Interviewer/ facilitator | Which author/s conducted the interview? | MSS was the main interview facilitator, AB, IC, and MM with prior experience in qualitative research, assisted the primary facilitator when translations from English to Mandinka (IC, MM) or Wolof (IC, AB) were required, and vice-versa. |
| 2. Credentials | What were the researcher's credentials? | MSS is a biomedical scientist, she was studying the MSc in Control of Infectious Diseases at the time this study was conducted (the work presented in the manuscript was part of the compulsory Project Report required to obtain the MSc). |
| 3. Occupation | What was their occupation at the time of the study? | MSS was a student of the MSc in Control of Infectious diseases, AB and IC were SpyCATS clinical nurses and MM a SpyCATS fieldworker. |
| 4. Gender | Was the researcher male or female? | MSS and IC as female, and AB and MM identified as male. |
| 5. Experience and training | What experience or training did the researcher have? | The three clinical research nurses (AB, IC, and MM) had prior experience in qualitative research facilitating interviews and analysing data. MSS had no previous practical experience in qualitative research and audited the LSHTM module *Principles of Social Research*. |
| 6. Relationship with participants established | Was a relationship established prior to study commencement? | Most participants had previously established a semi-professional relationship with the nurses of the study team that developed during the cohort study, but not with MSS. |
| 7. Participant knowledge of the interviewer | What did the participants know about the researcher? | Please see S2 Appendix for detailed information |
| 8. Interviewer characteristics | What characteristics were reported about the interviewer/facilitator? | Please see S2 appendix for detailed information |
| **Domain 2: study design** | | |
| 9. Methodological orientation and Theory | What methodological orientation was stated to underpin the study? | Following a grounded theory, data collection and analysis occurred iteratively during fieldwork. |
| 10. Sampling | How were participants selected? | A theoretical sampling approach was followed. Informants were purposefully and gradually selected based on emerging findings. |
| 11. Method of approach | How were participants approached? | First by the study nurses and fieldworker to enquire about recent sore throat episodes and to introduce MSS to the SpyCATS families. |
| 12. Sample size | How many participants were in the study? | A total of 19 individuals participated in the study. (5 semi-structured interviews and 4 informal conversations were conducted) |
| 13. Non-participation | How many people refused to participate or dropped out? Reasons? | One approached individual refused participation after consulting the compound’s chief. |
| 14. Setting of data collection | Where was the data collected? | All interviews were conducted in a quiet private area, elected by the participants, generally consisting of the compound’s outdoor communal area or the house’s living room. |
| 15. Presence of non-participants | Was anyone else present besides the participants and researchers? | Normally, no other adults were present. However, children would often come to sit around the participants and researchers or play around. |
| 16. Description of sample | What are the important characteristics of the sample? | See Table 1 in the manuscript |
| 17. Interview guide | Were questions, prompts, guides, provided by the authors? | See S1 Appendix |
| 18. Repeat interviews | Were repeat interviews carried out? | No repeated interviews were conducted. However, some participants were re-visited to clarify specific aspects of the interview after transcription. |
| 19. Audio/visual recording | Did the research use audio or visual recording to collect the data? | Audio recording was used to collect the data. |
| 20. Field notes | Were field notes made during and/or after the interview? | Some notes were made during the interviews and a structured interview summary was filled immediately after the interview. |
| 21. Duration | What was the duration of the interviews? | Between 20 and 40 minutes |
| 22. Data saturation | Was data saturation discussed? | Yes, thematic saturation was defined as when new incoming data produced little to no new information on the topic under exploration |
| 23. Transcripts returned | Were transcripts returned to participants for commend and/or correction? | No |
| **Domain 3: analysis and findings** | | |
| 24. Number of data coders | How many data coders coded the data? | One data coder (MSS) |
| 25. Description of the coding tree | Did authors provide a description of the coding tree? | Yes - See code book in S2 Table |
| 26. Derivation of themes | Were themes identified in advance or derived from the data? | Both |
| 27. Software | What software, if applicable, was used to manage the data? | Microsoft Excel and Word |
| 28. Participant checking | Did participants provide feedback on the findings? | No |
| 29. Quotations presented | Were participant quotations presented to illustrate the themes/findings? Was each quotation identified? | Yes |
| 30. Data and findings consistent | Was there consistency between the data presented and the findings? | Yes |
| 31. Clarity of major themes | Where major themes clearly presented in the findings? | Yes |
| 32. Clarity of minor themes | Is there a description of diverse cases or discussions of minor themes? | Yes – in the results section we present minor themes reported by single individuals and exemplify them with illustrative quotes |
