# Supplementary material for: Health-seeking behaviour and beliefs around sore throat in The Gambia: A qualitative study
Source: PLOS Glob Public Health. 2024 Mar 25;4(3):e0002257. doi: 10.1371/journal.pgph.0002257 (PMC10962789; doi:10.1371/journal.pgph.0002257)
Supplement: S2 Table — (DOCX) [file pgph.0002257.s005.docx]

## S2 Table: Code Book

| **Code group** | **Code Name** | **Code description** |
| --- | --- | --- |
| **Domain 1: Illness perception and interpretation** | | |
| **Case identification / symptomatology** | Verbal complain | Any mention of a child complaining verbally regarding throat pain or similar |
|  | Crying | Any mention of a child crying due to sore throat symptomatology |
|  | Lymphadenitis | Any mention of observation of swollen anterior cervical lymph nodes |
|  | Tonsilitis | Any mention of direct observation of exudate in the child's throat |
|  | Impact on eating or drinking | Any mention of child expressing disconfort when eating or drinking or caregiver observes the child having trouble eating or drinking |
|  | Fever | Any mention of fever or high body temperature |
|  | Cough & sneeze | Any mention of coughing or sneezing during the sore throat episode |
|  | Uvulitis | Any mention of observation of irritated or exudative uvula |
| **Perceived cause** | Poor hygiene practises | ﻿Any mention of sore throat cause that is related to any bacteria or poor hygiene practices among either the children or the people they live with |
|  | Food | Any mention of a sore throat episode linked with the ingestion of certain foods |
|  | Cold water | Any mention of sore throat being caused by cold water |
|  | Smoking | Any mention of sore throat being caused by smoking |
|  | Hand shaking | Any mention of sore throat that is related to hand shaking |
|  | Spiritual | Any mention sore throat that is related to *Jinns*, witches, vampires or foul winds |
|  | Exudative tonsils | Any mention of sore throat that is related to exudative tonsils |
|  | Uvulitis | Any mention of sore throat that is related to uvulitis |
|  | Unknown | Any mention of sore throat cause not being known either by the respondent or other people in the community |
| **Perceived susceptibility** | One single susceptible person | Any mention of only one person in the household reporting one or more sore throat episodes |
|  | More than one susceptible person | Any mention of more than one individual in the household reporting one or more sore throat episeodes |
|  | Children not susceptible | Any mention of a caregiver reporting that children never get sore throat |
| **Perceived frequency** | Recurrent | Any mention of reporting more than one episode of sore throat a year on the same individual |
|  | Occasional | Any mention of reporting one episode of sore throat a year, per individual |
|  | First episode | Any mention of a first household sore throat episode experienced being described during the interview |
| **Consequences/impact** | Pain | Any mention of pain being a consequence of sore throat |
|  | Impact on eating or drinking | Any mention of impact on eating or drinking as a perceived consequence of sore throat |
|  | Death | Any mention of death as a perceived consequence of sore throat |
|  | Lymphadenitis | Any mention of inflamation of lymph nodes as a perceived consequence of sore throat |
|  | None | Any mention of sore throat having no negative consequences or impact in the children |
|  | Trouble talking | Any mention of talking problems as a perceived consequence of sore throat |
|  | Missing school | Any mention of missed school as a perceived consequence of sore throat |
| **Sore throat awareness** | Concerned | Any mention of sore throat being a reason for concern for the caregiver |
|  | Non-concerned | Any mention of sore throat being perceived as usual or a condition that a caregiver should not worry about |
| **ARF awareness** | Heard about it | Any mention of claiming to know what ARF is or what are its signs and symptoms like |
|  | Known a case | Any mention of claiming to have known someone that suffered ARF |
|  | Never known never heard | Any mention of never having heard or never known anyone with ARF |
| **RHD awareness** | Heard about it | Any mention of claiming to know what RHD is or what it looks like |
|  | Known a case | Any mention of claiming to have known someone that suffered RHD |
|  | Never known never heard | Any mention of never having heard or never known anyone with ARF |
| **Domain 2: Health-related seeking behaviour and case management** | | |
| **Case management and health seeking narratives** | Traditional medicine only | Any mention of ﻿the treatment of sore throat with a combination of herbs, local physical interventions, other natural remedies, and spirituality. |
|  | Evidence-based medicine only | Any mention of the treatment of sore throat with biomedically accepted drugs |
|  | Combination of TM & evidence-based | Any mention of first using TM and then using evidence-based medicine |
|  | Societal change | Any mention of treatments or behaviours perceived to belong to the past and their reasons |
| **Reasons to attend health providers (Health centre, Hospital, farmacy or *marabout*)** | Severe illness | Any mention of attending the health provider due to sickness experience perceived to be severe or life-threatening |
|  | Sore throat | Any mention of attending the health provider for sore throat |
|  | Lack of drugs | Any mention of attending the health provider due to lack of drugs in another health provider |
|  | Time | Any mention of attending the health provider due to lack of time to attend another health provider |
|  | Specific disease for specific provider | Any mention of attending a specific health provider for a specific condition |
|  | Closseness | Any mention of attending the health provider of choice instead of others due to proximity |
| **Domain 3: Perceived barriers** | | |
| **Perceived quality of care** | Neglect | Any mention of an experience or observation of a neglectful event that can pose at risk the patients safety |
|  | Attitude | Any mention of a complain concerning the attitude of the healthcare workers |
|  | Lack of resources | Any mention of a complain concerning a lack of resources, such as drugs, at the health facility |
| **Costs** | Direct | Any mention of a complain concerning direct costs of seeking healthcare in a specific institution. Direct costs are monetary costs, out-of-pocket payments, and can concern drug purchase, doctor visit payments or transport costs. |
|  | Indirect | Any mention of a complaint concerning indirect costs of seeking healthcare in a specific institution. Indirect costs include long waiting times, lost time off-work or school. |
