# Supplementary material for: Health-seeking behaviour and beliefs around sore throat in The Gambia: A qualitative study
Source: PLOS Glob Public Health. 2024 Mar 25;4(3):e0002257. doi: 10.1371/journal.pgph.0002257 (PMC10962789; doi:10.1371/journal.pgph.0002257)
Supplement: S1 Data — (DOCX) [file pgph.0002257.s006.docx]

**Interview transcripts**

# P001

MS: Ok, so this is now recording

MM: hmm

MS: Thank you very much for agreeing, are you happy with it? [IC] So are you or MM going to translate?

IC: Can we both translate? I am writing on this so if he needs anything I can help

MS: Ok, perfect, thank you. Emm so can I know first how old is [p001 name]?

MM translates to Mandinka

P001 replies in Mandinka

MM: So fifty years, he says he has fifty now

MS: Ok, and how many children does he have?

P001: Four

MS: Four? Ok, and how old are they?

MM translates to Mandinka and p001 replies

MM: So the first son is sixteen years

MS: aha

MM: The second one if fourteen years.

P001:

MM: [third girl’s name] is thirteen years, and this one is eight (points at a boy)

MS: aha, and who else apart from the children live in this household?

MM: only him and his family

MS: So.. the wife also lives here?

MM: aha

MS: So which language do they speak?

P001: Mandinka

MS: hmm, and what education do you have?

MM: [ says Arabic school in the middle of the translation, P001’s phone rings and he picks up] he said he went to school but he do not complete school, stopped at Grade 6

MS: he stopped at primary?

MM: six

MS: aha, six

MM: Then yeah, he (not understandable) says something about work

MS: aha, ok. And then… when he finishes the phone call can you ask what does he work on? [2.44]

Only Mandinka language

Phone call finishes at [4.25]

MM: he is a driver

MS: a driver, aha, taxi driver?

P001: yes taxi driver

MS: ok, ok

MM: then he got eye problems, that is the time he stops driving

MS: ok so can he tell us a little bit about his sore throat experience?

MM: it is like what I told you yesterday

MS: can you translate? What he said?

MM: what he knows about it there is no medication for it, the only thing you can do is a compound which you are not eating from you can just go there and spit over there fence

MS: hm

MM: yes, or, [ Mandinka conversation] then you you use the, I don’t know, the mortar? yes, and you just lick it

MS: the mortar that you use for cooking?

MM: yes,

MS: Ok

MM: you just take it and lick it, so that one will also

MS: and this is the mortar from your house? Or from the other?

MM: yeah from your house

MS: and why would, can you ask him if, why would spitting to a compound where he won’t eat help get rid of the sore throat?

MM: because that is the belief, that is what they belief

MS: but can you ask him?

MM: that is the belief they have.

MS: ok ok

MM: He said now we stop that, so now if you have sore throat you go to the hospital

MS: ok, but he still does that?

MM: No, he said it has been a while since he get sore throat, just less than two days he got it and they given medicine

MS: and it stopped?

MM: yeah

MS: ok, and how worried were you about this illness?

MM: he said when he got sore throat sometimes he cannot eat rice, only drink milk,

MS: only milk?

MM: yeah

MS: ok, and did the children have the disease at the same time as him?

MM: no, children never get it

MS: children never get it?

P001: No

MS: ok, why do they never get it?

MM: then now we are in a modern civilization is here you know before what they believe is that the thing that cause sore throat is the people that are eating from the same plate or bowl, so if one of them just rub the hands on the fence or something that is what cause sore throat.

MS: ok

MM: So now people are more careful they just rub their hands.

MS: ok ehmm… so what, when he got the sore throat what did he do? Did you say he waited for two days or..?

MM: No no,

MS: Can you asked him what happened after he got sore throat?

MM: he said ehm, he thinks because you know before he smoke, they think because of smoking that is why he is getting the sore throat here, so but now he stopped smoking. Since they give him medicine he said before it is there is something inside the throat, but now he take out everything then after that the sore throat stopped

MS: ok

MM: yeah

MS: and how.. well, has he ever tried treating it at home? Is the treatment they would use at home the spitting or would they do like an infusion to help or..?

MM: they said before they used to do that, but now when he got it he just called our team who just came here to give him medicine

MS: so the thing that made the behavior change was the eh enrolling into the spycats, can you ask him?

MM: He said before he joined spycat when he got it he normally go to the hospital

MS: ok

MM: but eh… it is almost for five years he did not get it again until recently he started to feel it, but by then that is the time MRC was running the project.

MS: ok thank you, and can we ask a little bit about what do other people in the community say or think about sore throat?

P001 is telling off the children

MM: ok he said, he cannot say what other people think because normally he stayed at home he do not go out, so but, he knows that people have determined belief about sore throat, some people will drink something so that the sore throat will go away but some will do as he said and but for him, aha, after you know that was a long time but now he used to go to hospital for sore throat, so he can not say what other people think

MS: aha

MM: yeah

MS: can you ask him if he knows what does this drink contain? The drink that other people might use?

P001: says something about African medicinole

MM: He said sometimes there is this thing from the tree, it is not actually a tree but it is just like you know form of it is a short one and then people take it and boil it and you drink it. And then most people tell you to go and take that one boil it and take the sore throat away, so...

MS: thank you, can we know how long does it take P001 to get from home to the nearest hospital?

MM: maybe one hour, he said he would just go to the main road then take a vehicle and go to the sukuta health centre, so sometimes it can takes you hour.

MS: hmm and to go to the nearest pharmacy?

MM: 2 minute walk, he said the pharmacy is close, just a 2 minute walk. He said another one is here like this junction here, so that is less than two minutes walk.

MS: hmm, and to a traditional healer? Is there a traditional here around?

MM: no he do not know

MS: ok, and on which circumstances would he decide to go to the hospital or pharmacy?

MM: he said most of the time when he got malaria or he is sick, very sick, that is the time he normally go to the hospital or pharmacy, or if he have a very severe sore throat that is the time he would normally go to a pharmacy or hospital.

MS: hmm

MM: sometime he would normally go to the hospital to treat this throat

MS: ok so… it does not matter like the severity of how he feels does not determine where to go?

MM: so pharmacy, [Mandinka again] he said, if you see, when he is sick he would go to the hospital but if you see [17.48] he went to the pharmacy maybe he went to the hospital but not have the drug prescribed medicine for him and that is the time he would go to the pharmacy to buy it from.

MS: ok, ok, and who would normally see you at the health center? Hospital?

MM: he said he would just buy a ticket and go to the doctor, and doctor would check it.

MS: ok, and what would he give you for sore throat?

MM: he said cetirizine antibiotic

MS: cetrizine? Ok, and how do they diagnose?

MM: he said they give him something also, I don’t know, that will make your throat to feel cold if you take it, very cold

MS: ok, is it like a skin ointment or you take it?

MM: nono you take it, it makes your throat to feel cold if you drink a glass of water, but he can’t remember the name

MS: ok, and what type of diagnostic does the doctor do there?

MM: he said when he go there he would tell the doctor he is having sore throat then also after the doctor also confirm that he has sore throat.

MS: hmm, by touching, or looking or..?

MM: he said touching and also he put something inside you, he said when you have it here will be swollen and you can not swallow saliva, so the doctor will check and look inside also.

MS: and who pays for the healthcare?

MM: he says in the gambia you have to buy medicine for yourself

MS: ok, and how happy is he with the services provided in the..?

MM: some people have patience some people no patience [mixed with mandinka]

MM: he is saying you know sometimes you can go to the hospital and meet someone who is good and sometimes some of them are terrible, so if you meet those kind of people you will not be happy. So you meet the good ones here and it makes you happy.

MS: and what makes a good, eh, a good professional in his opinion?

MM: he said example if I meet you right now you just frown your face to me then I will not be happy maybe I will even be afraid to approach you, but if I come and you are smiling I can tell you anything I want to say. So that is what he is saying

MS: ok so we are approaching the end of it. Can I ask him if he has ever known someone with acute rheumatic fever?

MM: he has never known anyone because for him maybe this type of people you can find them at the hospital but for him he just goes there for his [unkwnown min 23.15] but he does not look around to see what other people are feeling or something

MS: and what has he heard about this condition?

MM: so eh he said sometimes this scabies maybe you can have fever or malaria than can give you those kind of fever, so he said those are the things he knows of.

MS: and has he ever known someone with rheumatic heart disease?

MM: no

MS: and has he heart about RHD?

MM: he said he had heard of it.

MS: and how does he know if someone has RHD? I think there is a miss-translation here as he is saying something like heart problemmo

MM: he said he can not tell but sometimes you will be sitting with people who said this person is having rheumatic heart disease,

MS; ok, ok, and why do they say it? Because they are feeling poorly or?

MM: it is their relative sometime

MS: ok so I have one last question, so before he said that when he was younger people could get sore throats from touching things and putting their hands in the mouth, and then he thought it was because of smoking and he quitted smoking, but now, if he gets a sore throat, why does he think he is getting it?

MM: he said ehh, sometimes you can have it to your friends, you shake hands with a person and you also have it.

MS: is there anything else he would like to say?

P001: no.. just thank MRC

MS: ok! Do you have any questions?

MM: so the question he has is that you know we come here with a project and ask them do you want to participate? And then when the project goes and finishes, everything is finish and it will if one of his children have malaria or something and you know he is not working because of his condition, so if he happen to have our number and call us for help to see if a child can have treatment are we going to be willing to do that?

MS: so… I have to ask Gabrielle, because I am not the doctor of the study, but I think that maybe I can ask the other doctors in Fajara what would happen then but I do not know because I am not a doctor

MS: abaraka bakeh

P001: thank you very much.

Part 2 of the interview recording

Date: 2/08/2022

MS: ok, so when he is done with the sampling can he tell us how did they do it and who did it?

P001 MM and E speak Mandinka

MM: he said they take a blade then tier this spot (he makes a sign of two vertical lines in both sides of the throat where the lymph nodes are are) then after they take needle and remove it.

MS: from here?

MM: yes

MS: so they cut here?

MM: no, cut it like this

MS: hmm

MM: then put needle there remove it, both sideways, then after there is pain, they put powder, then they put it there and tie it

MS: which kind of powder is it, do you know?

MM: no I don’t know

MS: hmm

P001 speaks Mandinka

MM: he said he can not tell the name, because at first people normally use this herbs to treat themselves, these trees in the forest or the small leaves and other things. That time it was the things they used to treat themselves.

MS: ok, and this was in Sukuta that happened to him?

P001: in upcountry

MS: upcountry

P001: yeah yeah yeah, (speaks Mandinka) after summer

MS: and who did it to you? Who took it out?

P001 speaks Mandinka

MM: I know that one is not alive now

MS: but is it a marabout or a traditional healer?

MM translates and p001 replies, E intervenes in conversation

MM: he said that time …

E interrupts and we continue at 2.53 of the interview recording

MS: so is it a marabout or a…

MM: no, he said, you know, those people are traditional healers, you know we have a lot of traditional healers before, not now,

MS: hmm

MM: before, not now

MS: so it was a traditional healer, ok

MM: and they believe that if you don’t remove these things, they are also part of the reason they start make people going crazy, mad, like that

MS: ahh

MM: so if you remove it you don’t have these kind of things

MS: ahhh ok, but what is it that…?

MM: but many people remove it before before, but now it is… people are not doing it

MS: and they remove it because of sore throat or just in case?

MM: sore throat and what I just said

MS: madness

MM: that is what they belief

MS: and you know… does he know if there is… sorry…. there is someone else doing it now?

MM: not now, now they are not doing it

MS: and in upcountry you can still do it? Or not?

MM: not even in upcountry

MS: even in upcountry, ok,

P001: speaks Mandinka

MS: because… why did they stop doing it?

P001: yoww… first time, first time people are good out, now, the civilization

MS: the civilization

P001: yeah

MS: and how long ago when you were a child this happened to you?

P001: only one week,

MS: when you were a baby?

P001: twelve years

MS: twelve years old, ok, ok

P001 speaks Mandinka to E

# P002

MS: so I am starting to record now, thank you very much for letting me have a conversation with you. Can you tell me a little bit about yourself to start with? Like how old are you for example?

P002: nineteen

MS: nineteen hmm, and with how many people do you live in here?

P002: sixteen

MS: and do you live with like brothers and sisters?

P002: yes siblings

MS: and someone else?

P002: mother and parent and step mother, and my brothers aswell

MS: hmm, are you married?

P002: no

MS: and no children

P002: no

MS: ok, and which level of education have you completed?

P002: just finished … high school

[phone call from gabrielle interrupts interview, I pass the phone to Amat]

MS: Sorry, it is the doctor, and which language do you speak?

P002: Mandinka

MS: So that’s everything, so now we can start with the sore throat related questions, can you tell me a bit about your experience with sore throat? How have you been feeling, when did you realize that you had it, what did you feel..?

P002: I don’t know how to say

MS: if you prefer to use them we can translate it is ok

P002: no… it does not normally happen to me

MS: hmm

P002: just sometime

MS: how often do you think it happens?

P002: usually when I am about to have a cough or sneezing

MS: how many times a year do you have it?

P002: maybe four or three times a year

MS: ok, and where does the pain start? Does it feel painful?

P002: yeah, here ( touches the throat)

MS: and how long do you like..

P002: normally 3 days

MS: 3 days

P002: hmm

MS: and how do you treat it? if you treat it?

P002: traditionally I use pepper

MS: what is it sorry? (phone ringing distracting the listening)

P002: pepper

MS: pepper

P002: yes

MS: and what is that?

P002: yeah, just lick it

MS: ok, and how worried do you feel when you have sore throat?

P002: I do not feel worried because it is normal

MS: normal hmm, and so would you, like, where would you get the pepper from?

P002: pepper?

MS: yes would you get it from the pharmacy? Or from hospital?

P002: no, home, yeah

MS: ok, home, do you make it at home?

P002: yes,

MS: and how do you prepare it?

P002: just powder, after you lick it

MS: so it is powder of a plant?

P002: yes

MS: and you mix it with water or?

P002: no, swallow it

MS: ok ok, so, you treat it at home?

P002: at home yes.

MS: and what else, do you seek help anywhere else, like your mum? Or brothers? Do you manage it on your own?

P002: yes

MS: and do you know what other people think about sore throat or do about sore throat in the community?

P002: no

MS: ok no problem. And can I know how far away do you live from a hospital?

P002: not too far

MS: not to far? How long does it take you to go there?

P002: hmm thirty minutes

MS: ok thirty minutes, and to the pharmacy?

P002: I would say 2 or 3 minutes

MS: ok, and in which circumstances or experiences would you seek for help in those places?

P002: when it stays

MS: when it..? (Unclear)

P002: when it stays

MS: when it stays for… how long?

P002: more than three days

MS: ok more than three days, ok… do you have any traditional healers around the area? Or..?

P002: I do not know any

MS: ok, and who sees you when you go to the hospital or the pharmacy? Who… ehh.. who attends you there?

P002: doctors

MS: and are you… like what do you think about the service they give you?

P002: its all right

MS: are you happy with the service there?

P002: yes I think so

MS: yeah? Do you think they help you with the sore throat if you go there?

P002: yes

MS: and how?

P002: they give me medication and help, hmm

MS: hmm, and is the medication you showed amat, was it given to you at the pharmacy or hospital?

P002: pharmacy, yes

MS: and who gave it to you?

P002: one doctor… but I don’t know his name

MS: and what did he do before giving it to you?

P002: hmm?

MS: did he ask questions, did he touch..

P002: hmm no

MS: did he look..?

P002: no

MS: just asked questions?

P002: yes

MS: and what are the medications that he gave to you?

P002: (gives it to me)

MS: is that antibiotics? Co-trimoxazole, and is that a syrup?

P002: yes

MS: and has it helped you to get better?

P002: yeah

MS: yeah? Ok perfect, don’t worry we are almost done. I wanted to ask you if you know anyone who has had acute rheumatic fever?

P002: No

MS: or if you have ever heart about ARF?

P002: No

MS: and about Rheumatic heart disease?

P002: no

MS: no aswell? You have never heard about it?

P002: no

MS: ok so is there anything else that you would like to say? Something we have not been talking about?

P002: no

MS: ok, and do you have any questions?

P002: no

MS: ok then abaraka for your time

P002: thank you

# P003

MS: So thank you to have an interview with me, can we start by telling me a little bit about yourself? Like, how old are you, which tribe do you belong to?

MM: /translates/

P003: I hmm I am about fifty, fifty years old, ok, and I speak Mandinka

MS: hmm

P003: originally our tribe is Serer

MS: which one?

P003: Serer

MM: Serer, hmm

MS: ok, and how many children do you have?

P003: hmm how many children I have almost five

MS: almost five? Is there one on the way? Or... like are they expecting another child?

MM: noo, it is like they can not just quickly said it is this amount that is why he said it is almost five

P003: no, it is five, five children

MS: ahh, ok, ok

P003: almost is six, but one passed away

MS: oh, I am sorry

MM: ah ok

P003: yeah

MS: and how old are they?

P003: huh?

MS: how old are the children?

P003: ahh children, first has ehh… twenty,

MS: hmm

P003: the second one… twenty-o-seven… almost twenty o seven help me (to Amat)

A: twenty oh seven?

P003: yes

They speak Mandinka

A: twenty twenty-two to minus twenty seven… ok that is fifteen years

P003: ok fifteen years

MS: so the second one is fifteen

P003: the third one is eh.. eight

MS: hmm

P003: the fourth one is five

MS: hmm

P003: then the fifth one is almost two years because one year ten months

MS: ok, hmm

P003: yeah

MS: and how many people live in the house?

MM translates to Mandinka

P003: two is not here, the elder ones are not here

MS: hmm

P003: seven

MM: there are seven in the house

MS: ok, and, what is... what education did you complete?

P003: finish

MS: ehmm.. like

MM translates to Mandinka

P003: secondary level, fourth form

MM: fourth form

MS: ok, and what is your occupation?

P003: constructor

MS: constructor? Ok, great.. ehmm.. So now I will move on to some questions about sore throat experience, yeah?

P003: hmm

MS: so... can you tell me a little bit about your child’s experience with sore throat?

MM translates

P003: ok… just I have only one girl who got I mean sore throat problem,

MS: hmm

P003: just her, eh… happen to her because my children do not complain about sore throat. But her… only to her... sore throat,

MS: hmm

P003: ok, mostly she (something not able to understand min 4.20) back to me with sore throat… almost I can say every year

MS: every year?

P003: every year, two or three times

MS: the same person? The same child?

P003: yeah

MS: only one child gets sore throat?

P003: yeah

MS: ah ok, ehmm and how do you know that the child has sore throat?

P: ehh she just explained it to me that she gets a sore throat

M: hmm

P: when swallowing eatings and a… eating she feel the pain at times, at times even giving a problem to her

M: hmm

P: that is the time I … I know and I consult someone… I mean doctors

M: hmm

P: even I am, I mean, if she explained to me and others (not understandable min 05.05) if injamou knows about it yeah,

M: hmm ok, and how long does she have it for normally?

P: hmm maybe if she used tablets maybe two three days go off

M: hmm

P: but later in the month or in the year she will get it again

M: ah ok

P: yes

M: and would the treatment be the same?

P: yes, the same treatment

M: ok, and what treatment is that?

P: it is giving ehh… some sort of tablets,

M: hmm

P: but I do not know the name of the tablet, I can’t say the name of the tablet, yeah..

M: ok, it is ok, ehmm and how did she get the disease? Or why? Do you know?

P: I have no idea of it

M: ok, ehmm… and how worried are you about this illness?

P: well, I am very worried about it, but I don’t, I don’t get anything to stop it but I mean I am trying my best

M: hmm

P: yeah

M: and why are you worried about sore throat?

P: ehh because I am very worried for her health wise,

M: hmm

P: you know, if you are not healthy you can not do anything, even to go to school she won’t go.. because of the pain… so I must be worried

M: hmm yeah.. hmm, ok, and do you think the illness can be dangerous for the child?

P003: well I don’t actually know very extended, but I mean, dangerous, but I mean prevent it is better than cure it. Maybe you do something before it goes harm

MS: hmm

P003: ok, that is why I got worried about it

MS: hmm

P003: yeah

MS: ok, thank you, and then you mention that after the child started to experience sore throat, what happened next? Which steps did you take? Did you first try to treat it home… or go straight into the doctor?

P003: No I mean I go straight to the doctor, no other treatment before because I do not have any other I mean method to take me, to use at home, just to go to the doctors,

MS: hmm ok, ehmm.. and who do you, like, are you in charge of taking her to the doctor? Or.. who makes the decision of… going to the doctor?

P003: normally I prefer going to the doctor first before doing any other thing,

Maha translates the question

P003: that’s is not our own mentality

MS: but the person who takes… goes to the doctor with the child is that you.. or the mother, or another person?

P003: at times she goes on her own, at times she goes with her sister

MS: aha, and who tells them to go? Or they decide to go?

P003: I decide

MS: you decide

P003: yeah

MS: ok, ehmm, and is anyone outside this house expecting you to also go to the … to seek for help?

P003: hmm no,

MS: ok, and do you know what other people in the community think or say about sore throat?

P003: I do not think the community thinks about sore throat… well I can’t tell more about that

MS: hmm

P003: but I personally experience it long long long time, not now, but I know how was it, because it can prevent you from eating

MS: hmm

P003: which is bad

MS: hmm, yeah

P003: drinking water, too

MS: hmm

P003: ok, maybe you get, I mean for the pain muscles and the joint pain too apart from sore throat so I know it is dangerous

MS: hmm, ok, hmm… thank you, and can we know how long does it take you to go to the health centre?

P003: hmmm

MM translates

P003: there is like thirteen thirteen mintues

MS: thirteen minutes? Hmm

P003: ehh yeah

MS: walking?

P003: yeah, by foot yeah

MS: ok, and to the hospital?

P003: yeah to the hospital and at times to the pharmacy

MS: and how far away is the pharmacy?

P003: the pharmacy is not at… much further just about ehh… ten to fifteen minutes walk

MS: ok

P003: yeah

MS: hmm… so… in which circumstances would you go … like you said before you always go straight into the … to see the doctor, would you ever decide to go to the pharmacy before you go to see the doctor?

P003: at times I go to the hospital direct, at times if I see that it is too late I go to the pharmacy.

MS: hmm… and what do they do at the pharmacy?

P003: the pharmacy… they … they do their checkings

MS: hmm

P003: before different tablets

MS: which kind of checkings?

P003: I do not know the name of your.. hahaha

MS: it is ok,

P003: usually tools other I mean…

MS: aha

P003: I see them, I see them in one room

MS: can you describe what do they do? Ehh

MM traduces to Mandinka

P003: (speaks Mandinka)… but I do not know what’s that.

MM: sometimes they would check on the sides, or sometimes they would open their mouth put something inside to check inside there is something or not.

MS: ok, hmm, and then they give like.. tablets?

P003: yeah

MS: ok, and in the doctor? Who sees you when you go to the hospital?

P003: Who sees me?

MS: yeah, or well the child, sorry

MM translates

P003: I can’t differentiate whether it is a doctor or not

MS: or a nurse,

P003: yeah

MS: ok, ok, and do you know what they do? Like the process is the same? Do they check like here..

P003: the same

MS: the same?

P003: yeah

MS: ok, ehmm, how.. like, what do you think about the service you receive in the health centre?

MM translates to Mandinka

P003: ehh well ehh… definitely I can, I can say more about that but I don’t understand,

MS: it’s ok

P003: I mean the treatment is good so it is good but I mean it can stop

MS: hmm

P003: because always she get it,

MS: hmm

P003: yes frequently but I can say every year, she get it

MS: hmm

P003: so just trying my best so but I don’t know where to get the fast treatment who can stop it totally

MS: hmm, ok, and the people at the health center are nice? Or… what do you think about the service they give you? Can you translate that?

MM translates

P003: some are nice some are not

MS: hmm haha, and what makes them nice? Or not nice?

P003: Some attend you very nicely and then talk to you, and then some are harsh, some deal with them very difficult

MS: hmm

P003: yeah

MS: and.. who pays fot the healthcare?

P003: I do

MS: you do pay

P003: yeah

MS: ok ehh… ok, we are almost finish. So now I would like to know if you have ever known someone with Acute Rheumatic Fever? Or if you have ever heard about it?

MM translates

P003: no

MS: and has he ever heard about it?

P003: well, (Mandinka speaking)

MM: he heard of it but has never experienced it

MS: hmm and what has he heard of it?

MM: he just heard that the disease is in the country

MS: ok.. and what are the symptoms?

MM translates

P003: I can’t tell

MS: hmm ok, it is ok, and have you ever known someone or heard about Rheumatic Heart disease?

P003: no, no

MS: ok, that is ok, ehmm, so… is there anything else that you would like to say?

MM translates

P003: that is going to be whether we have an assistant about this sore throat

MS: hmm

P003: yeah, where to, where to go and where to get a good I mean medicine for that

MS: aha

P003: because it worked but

MS: it worked…

P003: it worries me

MS: it worries you, well I think you are doing the right things because you are going to the health centre immediately and professionals are checking it, so I think it is good, but what you are doing…

P003: ok, yeah

MS: can I ask, now I am just remembering… is there any traditional healer around the area?

P003: traditional healer in this area here? No

MS: do you know if people go normally to traditional healers or…

P003: if they go to traditional healer I never go there

MS: ok, good, ehmm do you have any other questions?

P003: no

MS: no? ok then haha

P003: thank you

MS: abaraka bakeh this is the end of the interview, thank you very much I appreciate your time

P003: you are welcome

# P005

MS: So thank you very much for eh, agreeing to do this interview with me. Ehmm can we start by telling me a little bit about yourself? Like how old are you, how many children do you have…?

A translates to wolof

P_005: 24 years, no husband, no wife

MS: no children?

P_005 laughs

E interrups in Wolof

P_005: no children for now

MS: not for now, ok,

P: she is single

MS: ehh… ok, and, but , what languages do you speak? And which tribe do you belong to?

P: My tribe is wolof, and uh… she speak wolof, fula, jola and English

MS: ok, uhmm, and what education do you have?

P: she stopped at grade 8,

MS: hmm

P: and later she went to a hairdresser school

MS: aha, and are you working at the moment?

P: no

MS: no, ok, ehmm and how many people live in this house? And what is your relationship with them?

P: six

MS: six, hmm… and they are your siblings?

P: yes

MS: hmm and you are the oldest sister?

P: yes

MS: ok, good, so last week eh… you told me that there was one of your younger sisters who had a sore throat, right?

A: hmm

MS: ehh when did the sore throat start for your sister?

A: ehh more than seventy-two hours.. three days

MS: but when like, it started, like a month ago…two weeks ago…? Three weeks ago…?

A: she thinks a month ago

MS: a month ago, and the seventy-two hour is the…

A: symptoms

MS: the time that the symptoms last?

A: started

MS: hmm, and how did it end?

P: sometimes three days

MS: three days, so the eh.. how did you treat the sore throat? Like how did it stop? If they treated it

A: ah ok, they use warm water, and eh… black pepper, and took it

MS: ok, aha, mixed? Warm water and black pepper

A: yes

MS: ok, ehh… and how did you know that your sister had sore throat?

A: like eh… she would complain that there is something white like this exudates, eh… inside the throat and she can not eat drink, and so when she finally cannot eat or drink that is how.

MS: ah ok, so she saw the white things

Amat without translating: yes the exudates

MS: ehh… and how worried were you about this sore throat?

A translates

A: It is something normal for her

MS: aha

A: cause it usually happened

MS: to her?

A: aha

MS: and to the other siblings?

A: just to her

MS: only her

A: hmm

MS: and does this happen more than once a year? Or just once in a while…?

A: Sometimes, yeah (starts laughing cause there is a baby crying)… normally like every month, yeah

MS: every month?

A: sometimes not every month but some like it occurs some like… always occurs to her like ( then says something that I do not understand in min 6.07)

MS: aha… and she just has sore throat, no fever… or cough or…?

A: no

MS: just sore throat

A: yes

MS: and every time you take water and pepper and it cures?

She nods

MS: ok ehm… then did you, haha, so the last time you did not seek help from anyone, like, did she (the sister) come to you for help or did she… treat herself? Do you know?

A: the one she witnessed, like they did not call anyone, they just ehm… boil the water and eh… add some black pepper and give it to her

MS: ah ok, ok, that’s good, ehmm… and can you ask her if she was It like, anyone outside the household … (I get interrupted by a phone call that goes from 7.27 to 8.23)

MS: sorry, haha, sorry, so… eh… can you ask her if you know if there is anyone outside the household who is expecting them to seek for help for sore throat?

A: who? (he does not understand)

MS: anyone outside the household, like in the compound for example, that they expect them to do something about the sore throat? Or..

A: for them to do it..

MS: yeah, like, go to the doctor, pharmacy, or treating it at home…?

A: I do not understand the question…

MS: ok, like what does the community think about sore throats?

A: ah ok (and he starts translating)

P laughs a lot about a biscuit anecdote

MM: she said, eh.. some people do it like that you can buy a biscuit and you just eh.. rub it over here (throat) and then throw it there

MS: a biscuit?

MM: yeah,

GM: and then it take the pain

MS: ah ok… and which kind of biscuit?

MM: any type, you take it and then you can eat it

GM: because it is a good biscuit

MS: ah ok… and you can buy it anywhere?

She nods

MS: Ah ok, and do you believe that? Or..?

P: no

MS: does it work for the others?

P; Maybe

MS: maybe, ok, so can you tell us how long does it take you to go to the hospital?

A translates

A: thirty minutes

MS: thirty minutes… by foot? Or by another transport?

A: foot

MS: ok, and to the health centre?

A: so they prefer to go to the health centre than to the pharmacy

MS: they prefer to go to the… so the health centre is the thirty minutes and to the pharmacy?

A: the pharmacy is the walk

MS: so pharmacy thirty minutes?

A: yes

MS: and the health centre?

A: forty-five minutes

MM: for the hospital it normally takes 45 minutes

MS: ah ok, with car or like… taxi, or gelli gelli?

A: any, any of them

MS: ah ok, fine, ehmm and to the traditional healer? Is there any traditional healer nearby?

A: No

MS: she does not know?

A: no

MS: do you know if there is any in the neightbourhood?

A: no

MS: ah ok, and can you tell me when would you seek ehh help in the pharmacy or in the health centre for sore throats?

A: they would never go to hospital for a complain of sore throat

MS: ah, ok, so never to the hospital, never to the pharmacy?

P nods saying no

MS: ok, fair enough, ehmm. Ok, and then for other things who does normally pay for health care?

A: yes if they are sick.. she said when they are sick they go to the hospital

MS: but who… who pays for this spent?

A: they

MS: each one of the siblings? Individually? Or they seek money from…

A: yes

MS:… get money from the parents or…

A: yes the parents

MS: ok, and who makes the decision of going to the hospital? Or the pharmacy?

A: she is the one or the elder sister

MS: but who would make the decision? The older sister?

A: hmm?

MS: the older sister?

A: yes

MS: ok, eh… and who would go with the children to the hospital?

A: her sometimes or her (pointing at the older sister)

MS: ok so the oldest sisters?

P: hmm (affirmation)

MS: ok we are almost done, ehh… can you ask her if … have you ever heard of or known someone with acute rheumatic fever?

A: No

MS: no? and have you heard about this condition before?

A: No

MS: ok, and have you ever known someone with Rheumatic Heart Disease?

A: she said no

MS: ah ok they were different, different questions

A: ah ok,

MS: eh… so no for both conditions… and never heard about both conditions?

A: no

MS: ok, ok so… is there anything that you would like to add or ask?

A: no

MS: no?

A: no

MS: ok then… jerejef for your time, and that is the end of the interview

# P006

MS: Ok… so this is recording now… eh… I think I will have it here or… ah, ok, haha, thank you. So thank you [Participant 006 Name] for agreeing to participate in this ehh… in this study. Eh… we would like to ask, eh, first of all if you can tell us a little bit about yourself, like, how old are you? What do you work on?

P006: My name is [Name] eh… my schooling start primary school

MS: hmm

P006: primary one to primary six, primary six (not able to understand but probably the year he finished primary school) to enter secondary school from one to four

MS: hmm

P006: I finish my school eighty-seven

MS: in eighty seven?

P006: yes

MS: ah ok

P006: I start work in eh… in afric-quorn eh.. afrikan, it is a security… you do security there

MS: hmm, ok

P006: and I start there around… eighty-seven, I work there five months, and quit the job, because I have seen all the trouble (? Not sure), very difficult, seven to seven,

MS: aha

P006: I won’t do that, I have pursuit to go to Senegambia, that is year eighty seven eh.. maintenance site

MS: hmm

P006: I do maintenance there, eh… electric,

MS: ah, for an electric company?

P006: no is it the same eh… I work in an hotel, for their maintenance, anything broken we make that fix. And I’ll… do electric there for one, one year. Four months, I leave the place and now I go to, eh… Colon beach

MS: hmm

P006: I serve there about… I start eighty-nine, so I leave there two-thousand and seven.

MS: hmm

P006: I serve about sixteen years there, I went there I go to airport also,

MS: hmm

P006: one of my brother he is having a shop there, I work there about two years, I leave that place, I went to… lemon creek

MS: lemon…?

P006: Lemon creek

MS: creek? Ok

P006: is a hotel

MS: hmm, ok

P006: and I serve there for… almost sixteen years there also

MS: ok

P006: I left there this January twenty eight

MS: hmm

P006: now, I retired now

MS: you are retired?

P006: yes

MS: aha

P006: no more working to hotel anymore, I work myself now, I do afra, I call afra

MS: afra?

P006: chicken you know?

MM: chicken

P006: you see chicken, now I live, now that is my job now.

MS: ok, so they are a special type of chicken?

P006: no, not this one there, I work in a shops

MS: aha

P006: the cartinyons (not understandable 3.18)

MS: the cartoons?

P006: cartinyons you know for… chicken

MS: ah ok,

P006: chicken legs

MS: ah, alright, so you sell meat? The meat of these chickens?

P006: yes, that is my job now

MS: hmm… and how old are you now?

P006: ahh… might be fifty-two years now

MS: fifty two

P006: I born in sixty seven

MS: ah ok.. good, and which languages do you speak?

P006: Mandinka

MS: Mandinka

P006: yess

MS: and the tribe is… Mandinka as well?

P006: yes

MS: ah ok, aha, and… how many children do you have?

P006: six

MS: six children?

P006: yes, three boy three girls

MS: ahh ok, and how old are they?

P006: ehh.. I have twins

MS: twins?

P006: yes, boy and girl, she born nine… ninetyseven

MS: ah.. aha, like me!

P006: yes? Ahh! This is my boy

MS: ah, ok!

P006: name is [Name]

MS: aha

P006: [name] and [name] are there, this is my firsts ones

MS: aha

P006: [Name] and [Name] and [Name] and [Name], three boy three girls

MS: hmm

P006: yes, they are eh… eh… downroad is [name] + (uninducible), another one is there, twins also, boys or girls

MS: hmm, ok, and how old is the little one?

P006: two years now, two years and three months

MS: ok… ok, perfect, and eh… you said that one of your children had sore throat in… around May?

P006: Yes, [name-female]

MS: yes, [name of the girl]

P006: she is traveling now

MS: she is traveling… and how old is she?

P006: she is twenty and something years now, born in ninety-ninety seven

MS: ah ok, so twenty.. yeah twenty four-five

P006: yes, around twenty five or twenty six

MS: ah ok, and what was her experience with sore throat? What happened? Can you tell us a little bit more?

P006: I don’t know what she… she is having little, ehh… sometimes she said it, she is probably anybody (not sure) but never explained anything to me

MS: ok

P006: yes… last time these guys came here and take his bloods and eh… take it, the time you know, she is small and eh…

MS: so you do not know how it started? Like when did she start feeling pain?

P006: no no no

MS: or… did she had only pain here?

P006: pain yes

MS:… or somewhere else?

P006: might be here I don’t know but it might be here

MM translates

MM: he said he was complaining of eh… pain in the throat then we came here then took the sample and take it to the lab and check eh… and then confirmed it was sore throat

MS: ahh ok, so you got confirmation of a strep A infection?

MM: yes (without asking p006)

MS: ok, ok, ehmm... and how long did it… ehh… how long happened between the she complained and you seek for help?

P006: He never tell

MM translates

P006: two days I was here

MM: two days before he seek help

MS: hmm, and how worried were you about the sore throat?

P006 says something in Mandinka and by the end of the sentence says “I don’t know how to explain”

MM translates

MM: he feel like that when you have sore throat it always disturbing because it is difficult for you to eat or drink something, that is the worry

MS: so the worry is eating and drinking?

P006: hmm

MS: ok, ok, ehmmm… and did any other children have it at the same time as her? Or…

P006: no… she is normal and the children eat together, [name] and [name], the same bowl,

MS: and do the other children have sore throats?

P006: no no no…

MS: they have ever had sore throats, the other children?

P006: no no

MS: never

P006: never

MS: ok, and how do you think that she got the sore throat?

P006: well, I never experience that, because this…

MM interrupts in Mandinka and translates

MM: he said he does not know how she got it because even they were surprised

MS: they were surprised… so it was the first time she got it?

P006: yes, first time, but now it is ok now

MS: now it is ok, ok, eh… and did you try to treat it at home before calling them? Or..?

MM translates

P006: no no no

MS: no?

P006: no

MS: so you immediately called?

P006: yes

MS: ok, ehmm… and with the treatment? Did you get antibiotics or do you know which kind of treatment did they give you?

MM translates

MM: yeah he was given antibiotics

A: you tell me when you are ready yes?

MS: yes thank you… eh… so appart from them did you seek help somewhere else?

MM translates

P006: no no no

MS: so she took all the tablets?

P006: tablets, only tablets,

MS: and eh… until they finish the tablets or do you..

MM translates

MM: yes,

MS: yes

MM: yes until they finish

MS: hmm ok, and do you know what other people in this community do with sore throats?

MM translates

MM: just like what they told us yesterday, eh… you take hot water, then put black pepper there, then drink it

MS: take it, hmm, ok, and do you… do you do it? Ehh…

MM translates

MM: he says he knows about it because eh.. his grandparents teach him, yeah, this kind of treatment, but then if you have wound or or your leg is broken something like that…

MS: so it also works… the warm water with pepper also works for open wounds?

P006: no no

MM: he said you can treat all those kind of things

MS: ok

MM: he was taught how to do that

MS: ah ok, I am interested

P006: laughs

MS: can I… can you talk to me a little bit more about on what other things is this remedy useful for…? Eh, or did your grand, your grandparents told you… so it is good the warm… eh, sorry. So the warm water with the pepper..

P006: hmm

MS: you can take it for sore throat…

P006: yea

MS: joint pain

P006: yea

MS: wound?

P006: yea

MS: and something else or…? Do you know?

MM: no, joint pain is not that, you can not take the warm water with, that one is a different treatment, he said he learnt about all this things from the grandparents

MS: ahh… ok, so different tricks

P006: hmm yes

MS: for the other conditions

MM: yes..

MS: so the warm water with the black pepper is only useful for sore throat

MM: yes

MS: ok I get it now, thank you. Ehmm … and can we know, eh… do you know about what other people think about getting sore throat? Like how they get sore throat?

MM translates

MM: He said sometimes from the things you eat, just like eh… this eh… palm oil

MS: palm oil

MM: yes, palm oil, even me sometime if I take it it’s like ehh I kind of develop a sore throat

MS: yes?

MM: yeah, so there are certain foods that if you eat sometimes you can get it, that is what he said

MS: so this is palm oil and… do you know any other foods that can give you sore throat or that people say that give sore throat?

P006 speaks Mandinka

MM: he said sometimes if you take pepper, you eat pepper

MS: ah ok

MM: yeah you can have it, lot of pepper you can have sore throat

MS: ok… spicy pepper?

MM: yes, the spicy one

MS: ok hmm, thank you. Ok, so… can we know how long does it take you to go from here to the hospital? How far away is it?

MM translates

P006: half a mile

MS: half mile

P006: hmm

MS: and do you go walking?

P006: yes I walk

MS: hmm

P006: sometimes I take a car

MS: a car hmm

P006: yes

MS: and with the walking… how long does it take?

MM translates

P006: forty five or… forty three minutes yes

MS: ok, aha, hmm… and to the health center?

MM: health center is what we have around this area

MS: ok, forty-three minutes then, and to the pharmacy? Do you have any pharmacy?

P006: we have a pharmacy here

MS: just in front?

P006: yes

MS: so… one minute?

He laughs

P006: ha ha ha yes

MS: ok , so just around the corner

MM: is the pharmacy where A is working

MS ahh.. ok so you know him?

P006: yes hahaha

MS: and do you have any traditional healer eh… around?

MM translates

MM: he said there is none here except if you went then you must go to this urban areas, urban area around Basse, Brikama Ba… those areas, yeah

MS: ahh, so I’d had to go to upcountry?

MM: yes, to have traditional healers, he said around here, the majority of them are dead, they die now

MS: they are going?

P006: yes

MS: because people don’t go to them or…?

MM: no no, it means they are no more alive

MS: ahh, ok

MM: yes

P006: in sukuta you see see, but it is not much,

MS: ok…

P006: you can see some of them ran away, you know, they don’t want to take, you know, disease, this tris starts speaking Mandinka

MM: he said these are traditional healers, most of the time you know there.. the knowledge they have, sometime if they die, people are afraid to take them, so… that is how most of them are gone, many of this knowledge are gone now, people are afraid to take them. So that is why there are not much more now…

MS: so they are af…

MM: it is just fear of them, because I know of one but that one is around sukuta around that big mosque

MS: you know one?

P006 speaks Mandinka

MM: he can do it, that one, he will just recite something and tell the let it will go back to its possession

MS: ok

MM: because I did once go there when I have a new problem I went to the healer and he help it here

MS: hmmm

P006 speaks Mandinka

MM: he said even him, he can do it but he is not that much able to do it… he is afraid to do it sometimes

MS: but they are afraid of the treatment?

MM: no you know sometimes ehm… is just when you are doing this things you… is related to this eh… I don’t know if you know about this jinn, the superstition about the jinns and other things, so it is related to them

MS: what is this superstition?

MM: the spirits

MS: aha, and they are scared of the spirits?

MM: yeah, you know sometimes..

MS: of annoying them?

MM: no, you know sometimes it is given by the spirits like that, so if you are doing that sometimes is a problem

MS: ahh ok,

MM: yeah

MS: so are you afraid of the spirits?

P006: yeah sometimes,

MS: hmm

P006: mostly if you do… I can do close to my families, not, others no

MS: and how do you… can you keep them away?

P006: no, you can’t do that

MS: you can’t? ok, ok

P006 laughs

MM: that is why he only treat the family members, but not outsiders,

MS: aham… is that the traditional healer or him?

MM: him, he said he can do some of them

MS: so you are, you know how to treat it?

MM: yes

MS: and how do you do it?

MM laughs

MS: well… if you can tell

P006: you kind of check the… joints

MS: yeah

P006: and you push, you push, and can see they come back to the place

MS: ok

P006: yes I can do it in my children have problems if they have problems I do it

MS: and did you learn this from..

P006: from my grandparents, and grandmom

MS: and your grandfather was a traditional healer?

P006: yes, and my grandma

MS: ok, ok, and for sore throat this works?

P006: yes, all place it can work, but I did not do it, only in my family members

MS: hmm, ok

P006: or I know some of them, my friends can do all of them, but if not, I afraid

MS: hmm ok, ok, very interesting, thank you

P006: thank you welcome

MS: and can I know so… we have the health center close, the pharmacy, and you know a bit of traditional medicine, for sore throat, can you tell me in which circumstances would you use every different facility?

MM translates

MM: he said, sometimes you know the hospital is very far, he said half a mile, so and the pharmacy is close, so before he goes to the hospital, he just prefer to go to the pharmacy, instead, because that one is close to them

MS: ok, so first you go to the pharmacy and then… and what do they give you at the pharmacy?

P006: they check your blood, and… they check everything, after before they give you… tablets

MS: ok, hmm, but you said before that for sore throat you had eh.. just had one case of sore throat? Or before you would go to the pharmacy?... before spycats? Would you go to the pharmacy before this study?

MM: I don’t think they have any experience of sore throat before spyCATs

MS: yes, that’s why… I thought

MM: yes, I think it is spycat and then… that was the one

MS: ok, yes, fair enough, that makes sense… and in general, like, what do you think about, the people or the service that they give you at the pharmacy… and the service they give you at the health center?

P006: ehhh

MM translates

MM: he said, eh, sometimes this health centers, you can go there then you see nurses there then they would refuse to attend you they would be doing some other things. Sometimes you see a patient who is about to die or some patient had accident and they will just be careless until the person die. So he said but if you go to the pharmacy they will just attend you, if they see you they will attend you. You know the difference from pharmacy to the hospital is when you go to the pharmacy you are paying them, all the checkings and everything they are doing, you pay, then after they will sell medicine to you, but the hospital is different, sometimes you go there they will be chatting, whereas patients are there waiting, so that is what he said, so that’s why it is preferable to go to the pharmacy than to go to the hospital.

MS: ok, ok

MM: yeah

MS: and who pays for the medical care?

MM translates

MM: him

MS: you?

MM: yes

MS: and who decides when to go to the pharmacy or when to go to the hospital?

MM translates

MM: him

MS: you

P006: yes

MS: eh… ok, perfect, ehmm… that’s almost it… then.. I want to ask if you have ever heard about anyone having acute rheumatic fever?

MM translates and p006 telephone rings, he picks up and talks from min 25.17 to 25.46

MM: no

MS: not known anyone

MM: no

MS: and has he ever heard about the condition?

MM translates

MM: no, he never heard anything

MS: and about Rheumatic Heart Disease?

MM: no

MS: never heard of it, never known anyone having it?

MM: no

MS: ok, eh… so I have one last question, because I am curious… eh… the little one

P006 starts laughing

P006: [name of child]

MS: yes, [name] has this nice necklace, and the others… the others don’t, is it because he’s a baby? Or…? What is it for?

MM translates

MM: you know here… many people believe that when a child at its age… you know when they are teething they are just removing teeth sometimes is painful, eh?, so but if you put this in the neck it will not disturb him,

MS: yeah

MM: it will just remove all the teeth without any disturbance

MS: so it is for the teeth

MM: yes

MS: yes… in spain they do the same

P006: you are from spain?

MS: yes

P006: ahh ok

MS: and they have… some people put but with a different material,

MM: yes

MS: and I wanted to ask because I thought if this one is for the teeth?

MM: yes is for the teething

MS: so yes, ehm… that’s it from me, do you have anything that you would like to say that you think that I have not asked?

MM: translates

MM: he said it’s like you asked everything about health and other things so… he can not ask anything unless it is thank you

MS: ohh abaraka

P006: abaraka!

MS: and do you have any questions…?

P006: no…

P006 talks in mandinka to MM

MM: he said ehm.. since now the question he wants to ask is… since now they are joint to participanting to this project and it is like they are joint to us… so if somebody is sick or have an accident what are we going to do?

MS: while they are in the study?

MM: yes

MS: they have this on call telephone number that they can…

MM: call

MS: that they can call and eh.. one of the nurses is going to answer and provide the necessary help or come, right MM?

MM: yes

MM speaks Mandinka to p006

MS: ok then do you have any other question?

P006: no

MS: ok

MM speaks on the phone until 30.58

MS: he said to me that you were going to translate what he said in the end or something

MM: ah ok, he said ehm… they are very happy, the whole family is happy about that since they join this programme, every time they call if someone is sick they come so they are very happy

MS: ohh ok, so I will tell GC

They laugh

P006: thank you MS

MS: no, abaraka bakeh [P006 name], it was very nice speaking with you

# P007

MS: So this is recording now, eh… so thank you very much for participating, eh, in the study. Can we start eh… with asking you to tell me a little bit about yourself? Like, how old are you, how many children do you have…?

P007: I am thirty-three

MS: hmm

P007: I have four kids

MS: Four kids? hmm, and how old are they?

P007: This one is going to have eleven

MS: eleven, hmm

P007: this one is going to have eight

MS: hmm

P007: this one is going to have five

MS: hmm

P007: this one is going to have two years

MS: two?

P007: hmm

MS: now like in September or … soon?

P007: October

MS: hmm October, ok, and what is your occupation? What do you work on?

P007: I work at the gym site

MS: at the…?

P007: gym site

MS: at the gym! Ah ok

P007: yes

MS: ok, as a trainer or…?

P007: cleaner

MS: a cleaner, ok, hmm

P007: to clean many things

MS: ok, and what education have you completed?

P007: no

MS: no education?

P007: ah, eight grade, I stop at grade nine

MS: grade nine, ok, and is this like, which kind of eh… education is it? Is it like eh… national education or Islamic school…? Which system is it?

P007: school, Sukuta Junior school

MS: ok, perfect, and eh… what is the… which tribe do you belong to?

P007: Mandinka

MS: Mandinka, and you speak Mandinka?

P007: yes

MS: and English…?

P007: yes

MS: and any other languages or…?

P007: I can try Wolof also,

MS: you know a little bit of wolof, ok

P007: hmm, Fula also

MS: Fula? Ok, that is a lot! Ok, very impressive. Ok, so… did any of your children experience sore throat recently?

P007: only this one, sometimes she complained it

MS: ok

P007: yes

MS: and how do you… like when was the last time that she had a sore throat?

P007: long time now…

MS: hmm

P007: she complained it so I told it other group, I think that she complained two times

MS: is it like… more than one month ago?

P007: no

MS: one year?

P007: some days… it stopped

MS: hmm

P007: like me, is… the time I have it I use hot water

MS: hot water?

P007: yes

MS: hmm

P007: use it two days… it stop, all gone, so she also I did that, it stop cold water

MS: ok

P007: so i have it… stop cold water, only hot water in two days, it stays and three days then it stop

MS: aha… ok… so it was more than a year ago that she had it?

P007: no… not many months I think…

MS: only… a few months?

P007: three months old I think

MS: ok, hmm, and you had it a couple of weeks ago, right?

P007: yes, yes

MS: hmm, so the… how did you know that she had the sore throat?

P007: she told me, she said when I am eating it pain me, so I tell her open your mouth, let me see, twice, and look inside it, that one won’t break down (min 3.31)

MS: what did you see inside?

P007: nothing

MS: nothing?

P007: hmm

MS: hmm… and did she have pain anywhere else? Or other symptoms?

P007: inside throat

MS: did she have akademie? Here, in the head.. or?

P007: no

MS: no, only sore throat?

P007: yes

MS: ok, and eh.. you said you only gave water… hot water?

p007: yes

MS: without anything inside?

P007: no, no

MS: ok, and that it took… two days or three days to disappear?

P007: yes I think

MS: aha aha

IC interrupts and asks something about the house in Mandinka to p007. They have a short conversation and we continue at 4.36

MS: ok, ehm… and how long did it happen between she experienced the sore throat and received the treatment? The… hot water?

P007: yes

MS: did she receive it on the same day that she complained? Or wait a little bit?

P007: yes, yes,

MS: on the same day?

P007: yes

MS: ok, eh…

P007: at the time I was small, if I complained about this, my mother would take black pepper and go water small, put the black pepper small there, give you and drink

MS: hmm

P007: but you know the black pepper sometime... is hot, so I don’t use that, I only use that hot water

MS: hmm, ok, so you don’t use the black pepper because you don’t like it?

P007: yes, is hot, that is why

MS: it’s hot

P007: yes, yes

MS: ok,

P007: I use only hot water, I use that, stop

MS: hmm, ok, and why do you think that the symptoms of sore throat started?

P007: if I eating something I feel, pain me, if I swallow it

MS: hmm ok,

P007: I feel it

MS: ok, and how do you get it? How do you think you get the sore throat?

P007: I don’t have my mind there

MS: ok,

P007: just from eating, I think that is pain me

MS: but like… do you think you might have gotten it somewhere or… like, why do you have it?

P007: hmm

MS: you don’t know?

P007: I don’t know

MS: ok, and the other children?

P007: only this one, only this one

MS: and why do you think she has it? How did she get it?

P007: she also like me, she is complaining when eating to me.

MS: ok, and, are you worried about this illness?

P007: small

MS: small

P007: because it’s, it’s paining, if I do hot water it stop, that is why I don’t worry much

MS: hmm ok and when she has it do you worried about it?

P007: I use normally, that is why, if I see you people here complaining to them

MS: to them?

P007: hmm

MS: hmm, and can, do you think it can be dangerous?

P007: I don’t think

MS: no, ok, hmm and for the child as well?

P007: two years, is ok,

MS: and why?

P007: because if we have it we drink hot water, it stops

MS: ok, ok

P007: al right, I didn’t think it would be more dangerous

MS: aha, ok, ehmm… and appart from the hot water, did you tell anyone else?

P007: no haha

MS: and… do you know what other people in Sukuta or in… your neighbours here, do with sore throat?

P007: never hear anyone complain about sore throat

MS: ok, but do you know what people do? When they have sore throat?

P007: yes… they…

MS: can you tell me a little bit?

P007: they will be at… it is close to the hospital, some women, one woman is there, when you have it you will go there, she will use spoon and rub it black pepper there, when your tongue is full down, put it inside and make it stop…

MS: ok, and who does that?

P007: one woman, around the hospital

MS: is that a… marabou…

P007: no

MS: …or a traditional healer?

P007: yes, I think yes

MS: ok, a traditional healer

P007: yes

MS: and people around here go there?

P007: people around here they don’t know the person

MS: ok, and do you know him?

P007: yes, I know her, but that one died now

MS: ohh, ok…. A long time ago, or…?

P007: not long ago,

MS: ok

P007: maybe five months ago

MS: oh… ok, and it was a woman you say?

P007: yes, a woman

MS: aha, from Sukuta?

P007: Yes, sukuta, around the Hospital

MS: ok, and did you normally go there before she died?

P007: Our compound is there yes? Our compound is around there,

MS: ok, aha

P007: before we are there, so it was close

MS: and it worked? Eh.. the spoon worked for the sore throat?

P007: yes… yes

MS: aha, ok, and can I know how long does it take you to go from the hou- from here to the hospital?

P007: for me?

MS: like how long walking or… driving or… how long does it take you to go there?

P007: to go to the maribou or the hospital?

MS: both?

P007: when I have sore throat

MS: yeah, whenever you have to go

P007: well I go to the woman when I have sore throat there, I never go there, for myself, people with sore throat they stop their work they go there

MS: ok.. yeah, but I was wondering more about the distance… you know? From here to the hospital if I start walking, walking now from here to there how long is it going to take?

P007: I don’t… I walk

MS: ten minutes, thirty minutes, or…?

P007: I think thirty minutes

MS: thirty minutes, ok, and to the health centre?

P007: more than that, because there is so far, to walk, if you don’t have a car, you walk or you pass by and you know sign, until you take a car and go, and you walk small until you take a car.

MS: ok, aha

P007: cause me here, no car

MS: aha, ok, so… thirty minutes or forty minutes?

P007: more… maybe, this compound here

MS: here?

P007: one man, the one in the (something min 11) is there, he is going to the work around six o’clock in the morning, and I will prepare and go with him, to drop me around the hospital, so I go to the hospital, he is the one taking (childs name) when she is going to the school

MS: can you say that again? You said the one, who is taking her to the hospital?

P007: the school or bus, there is the man there, around six o’clock, he goes to the work at the Banjul, he is gone, then I want to go out and go to the hospital or I want to go somewhere, I inform her at the night, I call him at the night, so when the man is ready, the man will call me, I will wait, and he will take me,

MS: ok, ok… with a car I imagine?

P007: yes

MS: ok, good, and do you have a pharmacy… nearby?

P007: yes, around here

MS: aha, and how long does it take you to go there?

P007: maybe ten minutes

MS: ok, and then the… for the traditional healer?

P007: far, maybe thirty minutes, around the hospital

MS: but is there someone now or there is…?

P007: there is no one there now

MS: so you don’t have a… point to go?

P007: no

MS: ok… and, can I know like, in which circumstances would you seek help in the hospital if you have a sore throat?

P007: most people? me? This is my first time to have it, yes

MS: and you didn’t go

P007: no, but I told to these people, and they…

MS: ok, so you would normally… not go to the hospital for a sore throat? Just call them?

P007: yes

MS: and when would you call them?

P007: last time… other team were here so I complain about

MS: about it

P007: yes, these people,

MS: and what did they do?

P007: they told me that there is no medicine here, you can go to the hospital or pharmacy and buy there, when we come, we will return the money to you

MS: ok

P007: but before that I use hot water and it stop, so I did not go to the pharmacy?

MS: ok, so you did not go

P007: yes

MS: ok, hmm, have you ever before the study, gone to the pharmacy because of sore throat?

P007: no

MS: and for the children?

P007: no

MS: ok, and, ehm…. Apart from sore throat, like, what do you think about the… health workers or the… health center, are you happy with the services provided?

P007: yes I am very happy, I am very happy about you people because when my children sick I call you people

MS: hmm

P007: my children is sick, you care about it, give the medicine, and take care, it is very good

MS: hmm

P007: yes, I am happy

MS: haha, thank you, but then when you go to the Sukuta Health Center for example? Are they good?

P007: no, they will only give you the pass they will take it go and buy the medicine at the pharmacy

MS: aha

P007: or do whatever they like before they attend you

MS: hmm, what do you mean with whatever they like?

P007: you will go to the hospital you will see people very sick

MS: yeah

P007: they are not attained, they will do what they want

MS: aha

P007: after they will attend you and the children is very sick, not attend you, that is why I like MRC people, that time I was pregnant of this girl, I join MRC, but even there, I continue going there, until this girl has nine months, we graduate there, inside, MRC, inside

MS: MRC in Fajara…?

P007: no, sukuta

MS: ok, ok, ehmm… and who, who pays for healthcare in this house?

P007: my husband

MS: your husband, ok, and who goes to the health facilities with the children? If they are sick?

P007: me

MS: you go with them, ok, ehmmm… ok I think those are the main questions, ok, I also wanted to ask you if you have ever known anyone with acute rheumatic fever? Which I can translate to Mandinka… wait, it is here…. Balakando?

P007 speaks Mandinka and MM answers

MM: she said there is one kid here, around the compound there, who had it yesterday until he collapsed

MS: ohh… the acute rheumatic fever?

MM: yes

MS: ahh ok

P007 speaks Mandinka

MS: eh,… and what have you heard about this condition?

MM: what did…

MS: what has she heard about this acture rheumatic fever?

MM translates to p007 and she replies

MM: she said it is disturbing, you know, because even yesterday the kid was crying so she was the one who called a cab to come and take the child to the hospital

MS: hmm ok, so you where the one phoning

P007: yes because we don’t have car and, this man go to work, with the car, I know one drive I call the driver to come and take him, and go to the hospital

MS: ok, ok, and the child is in the hospital?

P007: yes yes he come back, he came back

MS: and is he better now?

P007: yes I think, I didn’t go there this morning

MS: ok, ok

P007: after I go and check it

MS: ok, and how do you if someone has this condition? Acute Rheumatic Fever?

MM translates and she replies

P007: I have never experienced it

MM: she says she has never experienced it, she just saw it yesterday

MS: but what did she see in the child, and she knew…?

MM: the body was hot

MS: hmm

MM: until he collapsed, and the mouth started to spitting this small sorts of wounds, something like that

MS: ok.. so by collapse what do you mean by collapse?

MM: loss of consciousness

MS: ok, ok, and.. have you ever known someone with Rheumatic Heart disease?

MM translates

MS: yusukusasa

P007: no

MS: no… ok, and have you ever heard about it?

P007: no, I have hear about it (continues in Mandinka)

MM: he heard that people got it but he has never experienced it

MS: ok… and what have you heard about the other people?

MM translates and p007 replies in Mandinka

MM: she said she just heard people saying it, but she has never witness someone that have it, or she has never seen someone that has it

MS: so she just heard the name or…?

MM: yes, the name like that

MS: … no symptoms?

P007: no

MS:… you don’t know how to recognize if someone is having it?

P007: no…

MS: ok, so I think this is the end of my questions… do you want to add anything? Do you think I have not asked something? Do you want to add something else?

P007: no, it is ok, thank you

MS: haha, no questions?

P007: no

MS: ok then thank you very much, abaraka bakeh!

P007: abaraka bakeh

# P008

MS: so thank you very much for participating in this study, is it ok if we start by telling me a little bit about yourself? Like how old are you, how many children do you have…?

P008: translator!

MM: ah, ok, [speaks Mandinka]

P008 replies in Mandinka

MM: So, her name is [Name p008], she is living in Nema, Nemasu, ok, she, she, has been to school and stop at secondary level, secondary fourth, then sit for some time, then after joined college

MS: college?

MM: aha, so now she is back to the school, to teach there

MS: aha, as a teach… you are a teacher? Aha, ok!

P008: hmm

MS: and what did you, did you study in college?

P008: what do you mean?

MS: did you say she went to college or…

MM translates

MM: just a teaching degree

P008: and I have five kids

MS: five kids? Nice, and how old are they?

P008: hmm… the first one… maybe help, maybe help me (asks Isatou)

E replies in Mandinka

P008: twenty-six, my eldest daughter is twenty-six, and the last one is twelve years

MS: ok, nice, eh… ok, and which tribe or ethnic group?

P008: I am a ganier (not sure how this is written)

MS: ah..?

P008: ganier

MS: ghanian?

P008: hmm, yeh

MS: ok, and which languages do you speak?

P008: I speak now Mandinka

MS: now Mandinka

P008: yes

MS: and other…? Like you are good in English as well

P008: yeah, I am not good in English, that is why I need him to translate

MS: she speaks very well though

P008: noo, I don’t speak, I just try

MS and p008 both laugh

MM: she is my teacher

MS: ehh… so mandinka… she was your teacher?

P008: noo! (laughing)

MM: no but she is my teacher now so let me just listen

P008 laughs

MS: ok, ok

P008: I will speak my language

MS: so… can you tell us a little bit about your child’s experience with sore throat? Who had the sore throat for the last time?

P008 speaks Mandinka

MM: that is the girl we talked to, at the tab there, her name is [Name] she said she had sore throat before, and she had it a lot, then she goes to the hospital

MS: hmm, and how log ago did she had the sore throat?

MM: hmm,

P008: not so long, because sometimes sore throat stop, it can stop quite a lot

MM: she said most of the time she do have sore throat

MS: ah ok, so most of the time means every week, or every month, or…?

P008: no no, not every week, maybe just time to time, you don’t know, we don’t even count, we can not predict

MM: you can not say, every week, but maybe… maybe it is monthly, weekly, some months before she got it back

P008: it is often

MM: so she usually have it time to time

MS: ok, ok, and she is the only child that has had sore throat?

P008: the eldest also used to have, for her her only is very serious, but she is not living here, she is married with someone

MS: ah, ok, and did she, how old is she now?

P008: [name]… she is getting to nineteen

MS: ok and when did she start having sore throats?

P008: no… I can’t remember that, but I know it is a long time

MS: A long time ago, so when she was little maybe

P008: yes, good,

MS: ok, and how do you know when she has sore throat?

P008: ok, sometimes, eh eh I am missing (laughs), you know, if she want to swallow somethings, so I see from her she can’t swallow, she feel tired, you know, cried like I can not swallow, neck is paining me, you know, from them I experience there is something wrong with her

MS: aha, so she has pain in the neck?

P008: aha, yes

MS: and somewhere else? Or just the neck?

P008: the neck, sometimes before having fever also it start with the sore throat, from I can… then she had fever

MS: aha… so first she has sore throat and then she has fever?

P008: she has fever, but not always, sometimes only the sore throat. But most of the times when she has sore throat she has fever too

MS: ok, ok

P008: hmm

MS: and what do you do when she has…?

P008: ok, sometimes, at first somebody told me we take eh… black pepper, put it in hot water, you know? and put it in the neck, she will not swallow the water but she would just leave it to the neck to be so hot. Maybe to drink hot tea… to drink hot drinks that will make it better, that is our belief, and some people also tell me that you should spit in the next… the compound you have never eaten before

MS: ok, ok

P008: you should stand at the fence, you undersand?

MS: yes

P008: then you spit, at their compound you have never eaten before, that is also our belief

MS: aha

P008: but those sometimes it never works, then we go to the hospital, I can remember sometimes they give antibiotic

MS: antibiotics

P008: antibiotics, I can remember that

MS: do you remember which kind of antibiotic is that?

P008: no… I can’t exactly remember but I know it is antibiotic, because I see the paper and antibiotic is written

MS: ahh ok, and when she takes antibiotics it stops?

P008: sometimes yes, but before it takes times, before it go off

MS: how long do you think it takes?

P008: sometimes… you don’t count when someone is sick,

MS: aha

P008: we don’t put that in your mind, you just see it going slowly slowly then is off

MS: aha, ok

P008: mr interpreter

MM: hmm?

P008: I miss it, [laughs]

MS: [name]

P008: is ok, let’s go [more laughs]

MS: [laughs] ok, ahmm.. and when she if she drinks the hot water with the pepper or the hot drinks how long, do you know how long does it takes? Is it faster than the antibiotics? Or…?

P008: the antibiotics are faster, this is just our belief, it is local belief, antibiotics are better than that

MS: hmm, and do you know what other people in the community do when the children have sore throats?

P008: yes, some take a spoon, I saw it,

MS: a spoon?

P008: a spoon, a small spoon, they will put these hot pepper there, you know? And put it in the neck, you understand?

MS: ahhh, inside?

P008: inside the neck, and it seems like you are vomiting, vomiting you know? You will be acting like somebody vomiting, that is our belief, then you said it is going off, but those are all…

MS: so… if you vomit, does the disease come out?

P008: aha, that is what we belief

MS: aha

P008: but sometimes it never works, but it is a belief

MS: aha, and who can do this? The mothers or the…?

P008: yeah not everybody can do it, but some eldest in the compound can do it

MS: ahh, ok so older people know how to do this

P008: yes they are the older people

MS: ok, that is very interesting, ehm… and what else? When she has sore throat and then you decide to go to the hospital, who goes with her to the hospital?

P008: sometimes, the first time I went with her to the hospital,

MS: aha

P008: but the others, she goes by herself, because when you go to the school with your uniform, it is easier to get medicine

MS: oh really?

P008: yes, when you go with your uniform sometimes you don’t follow the queue

MS: ahh ok , like why does that, why?

P008: they are students, because they think they are going back to school, so they treat them faster then they go back to the school

MS: so if they go alone and with the school uniform they get treated first?

P008: yeah yea

MS: ah, ok, ok, I didn’t know that, ehmm… and how long does it take you from here to the hospital?

P008: well… it is far, because we go to the Jammeh foundation

MS: ok

P008: yeah we take car from here to Latrikunda, then from Latrikunda we take another car, join another car to Bundung, we stop at the junction and we walk distance to the hospital

MS: ok, so, like..

P008: a little bit far

MS: so… one hour?

P008: yeah it can take one hour if you easily get transport, then one and a half hour

MS: ok, and to the health center?

P008: yeah I think that is the health center

MS: ok, so that is the Sukuta health center or…?

P008: no sukuta is here but Sukuta, I never go to Sukuta, I have never been there, I always go to Jammeh foundation

MM: the foundation is a hospital

MS: ah ok, to the hospital

P008: yeah it is a hospital, eh? Yeah it is a hospital, Sukuta is a health center, am I right?

MS: ok, yes that is correct, and do you have a pharmacy nearby?

P008: yeah yeah, many pharmacies around this area.

MS: and how far away from here are they?

P008: pharmacies are not far, I am surrounded by pharmacies,

MS: hmm

P008: yes, but I don’t go to pharmacies

MS: aswell

P008: yeah

MS: so… you have never been to a pharmacy?

P008: no, only maybe if I am prescribed a medicine for me then I can go to the pharmacy and buy it but I have never been there for treatment

MS: ok, ok that is useful

P008: hmm

MS: ok so you say you never go to the health center, you try the hot water eh.. and if that does not work you go to the hospital?

P008: yes

MS: and who decides when to do to the hospital?

P008: I decide it

MS: you decide, ok, good [laugh], and do you ever go to the traditional healer for sore throat?

P008: no no no no, ah! Yes! There was a time and when it… the first times it was really serious, I went to this traditional, they made, they take red rope, it is red, black and white, you know? Then they tied it at the neck

MS: ahh, ok

P008: maybe sometimes you see people tied ropes at their neck, it is because of that neck problem

MS: aha

P008: if you see anybody like that it is neck problem

MS: so it is red… black

P008: black and white

MS: is that called a Safou aswell or…?

P008: yes! Safou, it is called our Safou

MS: ok, ok, that is the traditional healer, and the marabout also…?

P008: yeah they are all the same, all the same, marabout and traditional healers I think they are all the same

MS: ok,

MM: no they are not the same,

P008: they are not the same?

MM: some traditional healers are not marabouts, but most…

P008: hmmm but most of them!

MM: Most of them are traditional healers

P008: most of them are marabouts because we believe in them

MM: most of the marabouts are traditional healers, some traditional healers are marabouts

P008: yes you are right that is true, he is very right, thank you for that

MM laughs and p008 laughs

MS: he is my teacher

P008: yeah he is my teacher too

MM: ahh noo

MS: ahh yess… and this safou is to cure the sore throat or to prevent sore throat?

P008: to cure

MS: to cure?

P008: to cure

MS: so first you have it, then you go, they put it on, and that is the cure?

P008: yes, we believe in those days we don’t even go, in those days people don’t even go to the hospital when you have this

MS: ahaa

P008: but now, is, you know… change of system people use to go, we believe in those, that safou around your neck, they will use hot water to prevent but that’s it

MS: ah ok, ok, good, ehmm haha, ehmm yeah, and then when you go to the hospital what do they do to the child? ehmm

P008: yeah sometimes when you go, you know she is a grown up, I don’t enter inside, at most, sometimes you go and you just explain what is wrong with her when you are out, they will go inside,

MS: ok but do you know if she is seen by a nurse or a doctor…?

P008: is a nurse, we always with nurses there

MS: a nurse

P008: hmm

MS: and what do you think about the service of the hospital

P008: ehhhmm it is good they try, they do a lot

MS: yeah? They treat everyone… ok?

P008: yeah

MS: ok, good, and who pays for healthcare?

P008: I pay

MS: you pay, ok… ehhmm and after you leave the hospital? Do they bring.. they… do they give you pills there? If they give you the receipt? Or do you go to the pharmacy right after the hospital? Or do you come back.. and wait a little bit?

P008: sometimes at the hospital there they give you some medicines, but some are not available, you know many people go to the hospital, then they would prescribe and write a medicine, then you find it, sometimes when I come I get money I buy it from they way coming, if not then I’ll come for some time, I get the money then… buy a medicine

MS: ok, so.. that’s almost the end… [she laughs] and one of the last questions is if you have ever known someone with acute rheumatic fever? And maybe for this… can we use a translation? Can you ask her if she ahs ever known someone with ARF?

MM: she knows

P008: nonono! she asked you to translate!

IC: that is a medical term! You don’t expect to know

MM translates and IC supports, p008 replies

P008: no, I don’t even know that sickness

MS: no, so you have not even heard about it before?

P008: no

MS: Ok, good, and have you ever known someone or have heard about Rheumatic Heart disease? And then we can use the medical term again

MM translates

P008: no

MS: no, ok that is fine, then do you have any questions? Do you want to add something? Do you think I have forgotten to ask a question that you think is important?

P008: no I think you have asked all questions hmm

MS: ok,

P008: perfect indeed

MS: ehhh, then that is the end of it, abaraka bakeh

P008: abaraka bakeh

MS: that was very good

# P009

MS: ok… so the device is now recording, thank you for agreeing to participate in this study… can we start the interview by telling me a little bit about yourself? Like, how old are you and how many children do you have?

P009: four

MS: four children, hmmm and how old are you?

MM: forty

MS: forty, aha, and how old are the children?

P009: this one year eight

MM: one year eight months

P009: six

MM: six years, seven years

MS: aha,

MM: this one is seven

P009 speaks Mandinka

MM: ah ok, so this is seven this is one year eight, then the two are not there

MS: ok, three boys one girl, perfect, ehmmm, and in the house, how many people live in the house? The children, with you…?

P009: yes

MS: husband?

P009: yes

MS: ok, ehhh… and what do you work on?

MM translates and p009 replies

MM: she is a house wife

MS: ok, ok, and the husband, what is the occupation of the husband?

P009: driver

MS: ok, and what education do you have?

P009: eight

MM: grade eight

MS: grade eight, ok, perfect, and what is the tribe? You are Mandinka or…?

P009: Mandinka

MS: aha, and speak Mandinka?

P009 says yes with the head

MS and a bit of wolof?

P009 says no

MS: other languages?

P009: no

MS: no, ok, ok, so… you said that there is one of the children that had sore throat?

P009: hmmm

MS: Which one is it?

P009 points at her, girl of about 12 years old.

MS: when did she have it?

MM: it take some time now

MS: hmm, like more than one month? Or…

MM translates

MM: one year now

MS: one year ago now, ok, and can you tell us what happened?

MM translates and p009 replies

MM: ok, she said, she is the one that complained that the throat is paining

MS: so.. you knew, how did you know she had it? Because she…?

MM: she said it

MS: she said it

P009: hmmm

MS: and what did you do after…

MM translates and p009 replies

MM: black pepper and hot water, and then she drink

MS: hmm, aha, and did the sore throat disappear with that?

P009: hmm (says yes)

MS: aha, and did you ask help to anyone?

MM translates p009 replies

MM: no

MS: no, ok, and why do you think she had sore throat?

P009 laughs because MM is distracted

MS it’s ok, he is the translator! He is the translator

MM starts speaking Mandinka and IC speaks to him in Mandinka, everyone laughs

MS: or IC you can also help us!

P009 replies in Mandinka

MM: because she said she can not swallow food, right, and also she can not drink water

MS: hmm, ok, but like, how did she get the sore throat?

MM translates, p009 replies

MM: she don’t know

MS: hmm, and do you know why? Is there a reason for which… is, does other children have sore throat? At the same time as her?

P009: no

MS: and before?

P009: no

MS: never

P009: no

MS: ok, so only her, one time,

P009: hmm

MS: ok, ok, ehmm, and then, do you know what the… sorry, were you worried about her having sore throat?

MM translates

MM: ok, she said how worry she was when she said she can not swallow food, that is the worry

MS: and do you think that the disease can be dangerous for your child?

MM translates

MM: yes

MS: yes, can you tell us why?

MM: basically because they can not eat

MS: ok, so those are the consequences

MM: yeah, and you can not drink, and if you can not eat for days is a problem

MS: ok, so that’s the problem

P009: hmmm

MS: ok, and do you know what other people in the community do when the children have sore throat?

MM translates

P009:no

MS: she does not know?

P009: hmmm

MS: and… how far are you from the hospital?

MM translates

MM: ehh… thirty minutes

MS: thirty minutes walking?

MM: hmm

MS: aha, and to the health centre?

MM: that is the health center, around here there is only one health center, that is Sukuta

MS: the Sukuta health centre

MM: hmm

MS: ok, and a pharmacy?

MM: it is not far from here

MS: like… five minutes?

MM: twenty five minutes walk

MS: ok, and do you have any traditional healer or marabout around the area?

MM translates

MM: yeah, they have a traditional healer

MS: aha, and when would you go to the traditional healer

MM translates

MM: she never go for that

MS: and why?

MM: ehh… the girl and she do not think it is that fun, or she… she belief in the hospital than in traditional medicine

MS: ok, and when would she go to the hospital? In which circumstances would she…?

MM translates

MM: when they have severe sickness, or ill, they would normally go to the hospital or health center

MS: hmm ok, and to the pharmacy? When would she go to the pharmacy?

MM translates

MM: here, majority of the people, the reason that take them to pharmacy is that when they prescribe medicine for them, and if it is not available at the hospital that is the time they would go to the pharmacy.

MS: ah, ok, alright

MM: it is not all pharmacies that check people and give them medicine, some of them are just there just to sell medicine

MS: ah ok, ok, so you first need to go to the health center

MM: health center yes

MS: ok yes, and what do you think about the service they provide for you at the hospital?

MM translates

MM: it is good

MS: aha, why is it good? Why do you think it’s good?

MM: she said it is good

MS: ok, and who decides when to go to the hospital?

MM translates

MM: the husband

MS: the husband, and who pays for it?

MM: the husband

MS: the husband aswell, ok, and.. how does he decide? Like, do you explain to him what has happened and then he says you go to the hospital or does he see first?

MM: no, you know sometimes, especially the drivers they don’t spent the all day here, if you see them it is plenty night time, so she would explain then the husband would say go to the hospital

MS: ok

MM: and give money

MS: ok, ehh ok, and can you tell me if you have ever known someone with Acute Rheumatic fever?

MM translates

MM: no

MS: ehh and have you ever heard about it?

MM: she said no

MS: aha, and about Rheumatic Heart disease?

MM translates

MM: no

MS: never? Never heard or seen anyone?

P009:no

MS: ok, so this is the end of the interview, do you want to add something? Do you think I have not asked something I should ask?

MM: no she does not have anything

MS: ok, and do you have any questions?

MM: no

MS: I have one last question… can I know how long did she have sore throat for?

MM translates

MM: four days

MS: ok, four days and then it disappears?

MM: hmm

MS: ok, abarakah bakeh!

P009: abaraka

# P010

MM speaks Mandinka

MS: yeah? So you are going to help me to translate?

MM: yeah

MS: ok so thank you for agreeing to participate in the study? Can we start by asking you to tell me a little bit about yourself? Like, how old are you, how many children do you have?

MM translates p010 answers

MM: no child for now

MS: no?

MM: she is not having a child for now

MS: ah, ok, ok

MM: she is 23 years old

MS: and do you have any siblings in the house?

P010: yes, I have a husband

MS: a husband? Aha, ok, congratulations [laughing] and ehm… does she live with someone else in the house? Do you have a…

MM: no

MS: a different compound?

MM: no, she lives here

MS: yeah, but does she live with the siblings here?

MM: no no no, she lives with the husband now

MS: ah ok, ok, then do you look after the other children?

MM: yes they are a lot of children around here

MS: ok, so and what is your ethnic group?

MM translates

MS: tribe?

P010: Mandinka

MS: Mandinka, and you speak Mandinka?

P010: yes

MS: and what do you work on?

P010: cleaner, [speaks Mandinka]

MS: where is..?

MM: sukuta, health centre

MS: ok! At the sukuta health center, ok, nice, and what education do you have?

MM: grade nine, she stopped at grade nine

MS: ok… that is with eh… twelve years old or something like this?

MM: no no, grade nine

MS: yeah but, how old?

MM: grade nine, sometimes some people, when they are grade nine, they are about fifteen, some are eighteen or nineteen, because from grade nine you are going to the secondary school

MS: aha,

MM: and then grade eleven twelve, and complete the secondary school

MS: ok, and have any of the children in the compound complained about sore throat?

MM translates

MM: no, but she is the one that normally complained of it

MS: normally you have it?

MM: yes

MS: hmm and when was the last time that you had it?

MM translates

MM: two months now

MS: two months ago?

MM: hmm

MS: and what happened?

MM translates

MM: she said when, when she has it, the symptom that she normally see is, the pain will start from here (touches both sides of the maxilar) it will arrive right up to the ears, so she can not eat or drink something

MS: ok, so the pain started on the sides of the neck?

MM: from here, yeah, went up to.. she can even feel up to the ear, inside the ear.

MS: and di you feel something else?

MM: the body, she used to have fever too

MS: ahh, ok, ok, and what did you do?

MM: she take medicine

MS: which medicine?

MM: paracetamol

MS: paracetamol, ok, and it went.. it was ok?

P010: no

MM: she said when she start taking, before the sore throat goes away, it take some time

P010 speaks mandinka

MM: she said there is one Marabout around here that will make this safou for her, and she will just tied it on the neck.

MS: aha, and that was, that’s what you use?

P010: yes, that’s what I use

MS: and it worked?

P010: yes

MS: ahh ok, and were you worried about the sore throat?

MM translates

MM: it used to disturb her a bit,

MS: sorry?

MM: the sore throat, used to disturb her, she can not even do her works, her chores at home ehh

MS: aha, so you are concerned about it?

MM: yes she said she is concerned about it

MS: do you think it can be dangerous?

P010 replies in mandinka

MM: she said she don’t know in the future but right now, when she has a sore throat, the way the sore throat is disturbing her she knows it is dangerous

MS: ok, hmm, because why is dangerous?

MM: the problem is… you can not eat

MS: hmm can you ask her why does she think is dangerous?

MM: because of the eating

MS: because of the eating

MM: yes, she can not eat or drink something, and she can not do anything, so she would just lie down like that, with the pain

MS: aha, ok, ok, and the… why do you think you get sore throat?

MM translates

MM: she said when she eat fish

MS: fish?

MM: yes, that is the time she gets sore throat

P010 speaks Mandinka

MM: or if the children eat, then after they wipe their hand with, on the wall or the fence or something that is the time she had it

MS: ok, and the children never have it here?

MM: no she, they never experience it

MS: they never complained?

P010: no

MS: ok, and how… like, do you know what other people in the compound do when children have sore throat?

MM translates

MM: she said eh… her mother used to do it, when she had sore throat they will take this black pepper, then they… she will put it in a spoon and you see… only the tongue that small, you see, then, she will use that spoon to put that up, so she will do it in the morning, afternoon and evening time, for three days, then the sore throat will go

MS: ok, and they still do… they do it now?

MM translates

MM: yeah, under the throat,

P010 speaks Mandinka

MM: ah ok, inside the throat

MS: ah ok, and rub it? In the throat? Ok ok, ehhmm, ok, and how far away are you from here to the hospital?

MM translates and p010 replies

MM: it is not far

MS: is not far? Like… walking?

MM and p010 talk in Mandinka

MM: less than 20 minutes

MS: 20 minutes, ok, ok, and that is the health center as well?

MM: that is the health center sukuta, around here is only sukuta health center

MS: ok, ok, and the pharmacy?

MM translates

MM: the pharmacy is here, when you are outside the compound you have the pharmacy

MS: So one minute…?

MMS: yes

MS: Ok, and the marabout? How far away is it?

P010 speaks

MM: she said less than 10 minutes away

MS: ok, and the one here? Is it a marabout or a traditional healer?

Mm translates

MM: yeah, it is a marabout

MS: aha, and why does she go somewhere else?

P010 speaks

MM: ok, she said eh… to have medicine from him is difficult, you see the queue?

MS: yeah

MM: yeah, so what she said that there is this powder thing that he normally give you, so you will just boil it, you know? Put a blanket on… cover yourself then open your mouth so that the flame of that hot water will get inside your throat

MS: ahh

MM: yeah, so then something will come out then after it will go away, but it will take one year before you have it again

MS: one year before you have it again?

MM: yes, but to have it from him is a problem, because (not able to hear what he says min 8.49)

P010 keeps talking

MM: she said, she said you would normally go to the bush to look for that

MS: to the bush

P010: yes

MM: so that is why to have it is difficult, that is why they are not having it here

MS: ok, thank you, sorry, ehmm… and… and do you know the name, the name of this plant?

MM: sorry?

MS: do you know what’s the name of this bush?

MM: no

MS: the name of the bush?

P010: no

MM: ok, no she does not know the name

MS: ok, and can we ask her, when would she go, when she has a sore throat, when would she go to the eh… traditional healer, for example, or marabout?

MM: when she has it she would normally go to there

MS: so first she goes there?

MM: yes

MS: and would you ever go to the health center for sore throat?

P010 replies

MM: no no, she only go to the marabout

MS: and pharmacy?

P010: no

MM: no

MS: no, for paracetamol, maybe? Ok

P010: yes (Mandinka speaking)

MM: only for paracetamol for the pain, then she go to the marabout

MS: ok, and who pays for this?

P010 speaks Mandinka

MM: she pays

MS: ok and when did you start to have sore throat?

P010: since I am young, like this this girl, because my mum used to take me since we are young we are getting this thing

MS: yeah, and did your other siblings also get it or not?

P010: no

MS: just you?

P010: yeah

MS: ok, and who did they also take you to the marabout, your mum? or would she do the pepper, black pepper thing?

P010: no that is my mum,

MS: ok

P010: my mum used to do that

MS: ok, ok, eh… and, ok, and then, have you ever heard about Acute Rheumatic Fever? Or known someone who has it?

MM translates

P010: no

MS: no, have you ever heard about it?

P010: no

MS: ok, and about Rheumatic Heart disease?

P010: no

MS: never heard about it, never known someone?

P010 says no with the head

MS: ok, so this is it, ehh… do you want to add anything else? Do you want to say something about sore throat that you think is important?

MM translates and p010 replies

MM: no

MS: no? ok, do you think there is a reason for it to come back to you?

MM: because of eating fish, it is the fish

MS: it is the fish

P010: I stop it, I stop it

MS: but… and two months ago did you have fish?

P010: no I don’t even eat it now

MS: and why did you have it you think two months ago?

P010: if I know it is when I eat with the childs, they wash their hand already after doing their hand like this (rubs it against the wall), I have sore throat

MS: ah, ok, it makes sense… do you have any further questions?

P010: no [laughs]

MS: ok then enjoy your meal! Abarakah bake

P010: abaraka bakeh [more laughs]

# P011

MS: thank you for the participation in the study

MM translates

MS: can we start the interview by telling me a little bit about you? Like how old are you, how many children do you have?

MM translates, P011 replies

MM: fifty years old

MS: hmm

MM: nine children

MS: and how old are they? Or the youngest and the oldest?

MM translates

MM: she can not remember the age of the eldest

Mandinka conversation

MM: she is calling the daughter to come and tell the age, but the oldest she does not remember

MS: it is fine, and eh… how many people live in the house?

MM: five

MS: five, ok, and…

P011 tells off some of the children in Mandinka

MS: and what do you work on?

MM translates (he translates after every question if I do not specify otherwise)

MM: gardening

MS: Gardening?

P011: hmm

MS: ehh… for someone or do you grow vegetables?

MM: no, on her own she grow vegetables in a small garden like that

MS: aha, do you sell them in the market?

MM: yeah when they are like… she normally grow and sell them

MS: ok, hmm, hmmm, and which education do you have… like in the school?

MM: she was going to Arabic school

MS: Arabic school

MM: but it is not far

MS: it is not…?

MM: just like a [unintelligible] it does not have grade

MS: it does not have credit?

MM: no, they don’t have grade

MS: ahh

MM: they are not in grading system, you just go

MS: aha

MM: it is a big hall, everybody will just sit together, then the… your teacher will be giving you what to read

MS: ok

MM: yeah

MS: and how many years did you go?

MM: no she said it is not for her, just a little bit then after she stopped

MS: aha, ok,

The girl daughter arrives

MM: she is the last…

MS: so the oldest one?

I heat the girl say “14” assume that is her age

MM: no, she is the last child

MS: ok

MM: the child, the last child

MS: So this is the youngest the little one

MM: yes

MS: and this one?

P011: ah ah

MM: no no, it is the, they are the grandchildren of them

MS: ahh

MM: their father is the oldest son now

MS: ahh ok, ok, and they all live here?

MM: yes, hmm

P011: hmm

MS: ok, ok, nice, ehh… ok,

MM: they are grannies, they are her grandchildren

MS: nice, good grandma [laughs] and which tribe do you…?

P011: Mandinka

MS: and language?

P011: Mandinka

MS: aha, do you also speak wolof? Or…

MM: sometimes they can, they speak wolof

MS: ok, nice, eh… and who was the one who had the sore throat more recently?

MM: she is the one

MS: she is the one who got it most recently had one? Ok, and can you tell us what happened?

MM: she said she wake up one morning and said ehmm this side is paining, then what she did was she just boil hot water and she is continued drinking that hot water the whole day but they see it is not better, then that is the time they took her to the health center, Sukuta

MS: to Sukuta health center

MM: yes

MS: aha

MM: so they give her medicine

P011 speaks

MM: ok, and they give her some medicines, then when she starts taking them, before those medicines finish, then the sore throat goes away

MS: ok, and did they… when the sore throat goes away, did they stop giving medicine?

MM: yeah, she did not take the medicine again,

P011 speaks mandinka

MM: ok, she said after that before she gets sore throat again it takes time, it take some time

MS: hmm, how much time?

MM: almost a year

MS: almost a year, hmm, and did you… when you gave the hot water, was it only hot water, or did you use pepper or lime or something?

P011: water

MS: water, hmm, ok, hot water, and hmmm

MM attends a phone call and we return to it until 6.27

MS: I was asking her if she was worried about the sore throat, or how worried she was?

MM: similar to the study, if you have it you can not eat or drink, so that’s always their worry

MS: hmmm, ok

MM: so she said, when she start taking medicine, then she has to take hot food, yeah, if she is eating and drinking anything it will be hot

P011 speaks

MM: what she is explaining is, you know in their tradition, eh… Mandinka’s tradition, they believe that what cause sore throat is eh… when you open your mouth this small, eh… it is just like a small thing, it is like a tongue, inside, so sometimes it will just collapse like that

P011: hmm

MM: so the place where it was lying, if it takes three days sometimes, you don’t put anything, that place might have something like a wound, or after it will swollen, then maybe even have this pus inside, so but what they do is they take a small spoon, just like what that lady explained last time, a small spoon then they will put black pepper there, so that black pepper they would open your mouth and put it so that they can bring that thing up, you understand? so that it will not be infected, because if it infected it can kill someone, that is what she said

MS: but then the spoon is used to take the … the white thing out? Or..

MM: yes

MS: or to do like the… [makes gagging sound]

MM: so they will just use the small spoon, with the black pepper to make it stand again

MS: eh… ok, so the stand again is the thing in your back… like the…

MM: yes

MS: … in the side or at the top?

MM: no, at the top

MS: ok, ok

MM: that’s it, so the black pepper and spoon will just help, help you to put it up so that it will stand again and the sore throat will go away

MS: ok, and did they do this to her that time?

MM: she once did it to her

MS: she… once?

MM: once, yeah

MS: when… like, how long ago?

MM: almost three years now, she last did it

MS: and when would she do that… like why three years ago did she do this but this time they went to Sukuta health center?

MM: ok, she said they… the oldest son, the father of these kids, when he had sore throat, he normally go to the health center, so that’s why she also decide that I’ll go to the health center this time, since that one go there, it will stop, so I will also try

MS: ok, because eh… because the health center was quicker than the, the other technique or because of the younger children telling her to do it… or why?

MM: ok, she, she… she said that ehmm, the reason is, you know when you go to the hospital, or sometimes especially children or big… even, even elderly person, people, sometime if you want to put spoon inside their throat they will not accept it, it is difficult, some will not allow to open their mouth so that you can… do that, so that is why they see that going to the hospital since they see that the tablet is just swallow, is more easier

MS: ah ok

MM: yeah, and she said that because of this refusal of putting a spoon there was man that did pass away because of that because the thing… the thing… the mouth was not going to the hospital but he is refusing to put the spoon inside his mouth

MS: wooow, ok, ok, and eh… do the other children also get sore throat?

MM: No no, nobody have it

MS: so is always one… like one girl, one time a year?

MM: when she had it before, before it come back again it takes so many time

MS: ok, ehmm… and how do you think… why do you think she gets it and the others don’t?

MM: she didn’t know how she get it… she does not know how she get it, so the only time she would know is when she complain of this side is paining, then after she will not be able to swallow, and take food, drinks and other things, that is the time she will notice she was having a sore throat

MS: hmm

MM: so they have this belief that if you take cold things it will worsen the sore throat, so you only take hot things, that will get… make it better very fast

MS: ok, aha, aha, so there is no, no cause?

MM: no cause

MS: ok

MM: they don’t know what is the cause of having that thing in the sore throat

MS: ok, ok… ehmmm and what do you on what other people, like your neighbours do with when their children have sore throats?

MM: she said the neighbors around she never had anyone complain about sore throat

P011 keeps speaking

MM: she she… she has never heard of them complaining of sore throat, because they are not, they have not been here for long, it is just three years they have, so before she was not in this area

MS: ok… and can we know how far away are you from the hospital?

MM: it is not up to 30 minutes

MS: 30 minutes walk?

MM: hmm

MS: ok, and the pharmacy?

MM: 15 minute, because that one is only there

MS: ok, and when would you go to the pharmacy?

MM: what she is saying is ehh…. When ehh the children maybe someone from them is complaining, or she sees that they have fever… a little bit fever, so if she is not having any time to go to the hospital, then she will just go to the pharmacy, then when she go there they will make some teses from them, test like taking the temperature and the RDT malaria RDT, then after they will tell her either they are positive for malaria or negative, then from there if you are positive they will give a quatern(?) the treatment of malaria, if not then they will give her other drugs, then she will come back and give them to the children, then two three days that will go off,

MS: hmm,

MM: they will get better

MS: ok, does she ever go to the pharmacy for sore throat?

MM: no, (…) she said once, (…), ok, they went to the hospital and they prescribe medicine for them, then she went to the pharmacy to buy them.

MS: ok, ok, so normally first to the hospital and then… the hospital is sukuta health center in this case?

P011: hmm, yes sukuta health center

MS: aha, you always go there?

MM: is closer to them

MS: ok, than the hospital

MM: the Jammeh foundation is very far

MS: aha, and in the health…

P011 interrupts in mandinka and speaks for a long while

MM: ok, what, what she is saying is ehmm… you know, eh… sometimes going to Jammeh foundation is very far, because you have to pay fare to go there, but here is closer to them, you can walk and go there, so, but eh… sometimes the difficulty is that when you go to the hospital you will join the queue until you reach them there, they write a paper for you, say go to lab, then you go to lab then you do the test, after you come back, then they will tell you the medicines are not here so you have to go back pharmacy and buy them, so that is why sometimes if you have money, they prefer to go to the pharmacy because when you go you will get all the test there, so they will just check you and give you the drugs

MS: hmmm

MM: but the problem with pharmacy is that they will not know whether you, your hepB is low, they don’t use check that, most of the times they just check your malaria then BP, but they will not check the hepB, and also if you are… you know, if you are not having enough ehh… maybe your, you need fluid, for example, normal saline or other things they will not realize that won’t,

MS: hmm, ok

MM: yes, so that is why sometime hospital realizes those things but pharmacy, if you have money they are the ones that have good drugs that you can… help yourself with

MS: hmm… the pharmacy is more expensive?

MM: yes it is because they sell everything, even the test they do to you, you pay them, even the RDT you pay,

P011 speaks Mandinka and says something on malaria test and 50 dalasi

MM: yes, you pay the RDT, any test they do, you pay, then after they will sell the drugs to you

MS: ahh ok, and in Sukuta?

P011 replies in mandinka

MM: if you go to sukuta, you buy ticket, so the ticket is 25 (something unintelligible at 23.20) is the same thing, but when they are doing this analysis for you, you pay,

MS: ok

MM: that one I think is 50 dalasi, if not you don’t pay anything, the other tests are free,

MS: aha

MM: but sometimes the medicine is the problem

MS: aha

P011: gabou gio (something else in mandinka)

MS: can we ask her who sees her at the pharmacy and at the hospital? Like…

MM (without translating to her): pharmacy… anybody, sometimes eh… people just take people and you know, take them to the pharmacy to work for them, sell drugs there, so sometimes some nurses will be around, those are the ones that will do the checking for you

MS: so the checkings…

MM: you know, you know, the majority of these pharmacies, you just go there with your prescription that was given to you at the hospital, then they will check the drugs that are there, they will sell it to you,

MS: and in the sukuta health center? Are you seen by a doctor or a nurse or…?

MM (now asks p011): only the serious, the serious illness those are the people that are seen by the doctor, but these other people are seen by the nurses

MS: ok, and when the children have sore throat in the hospital? Who sees them?

MM: a nurse

MS: a nurse, and what do they do?

MM: she said they will just eh… light, maybe either their phone or something, then you open your mouth, they look inside, just see how it is and they will write their findings like where it is paining and how it is paining

MM’s phone rings MS asks if its IC and MS says tell her we are almost done 25.43

Return to interview at 26.00)

MS: so what did she said in the end? They put a light and then…

MM: yeah, they look inside then they will ask you where it is paining and all the thing they will write their findings,

MS: and do they always give medication or sometimes no?

MM: then after they write everything they will ask you to go to the pharmacy then you get dispensed the drugs to you

MS: ok, ok, and… can we know if there is any traditional healer or marabout around here?

MM: they don’t have it around here

MS: ok, but would you go to the marabout or traditional healer for a sore throat sometime?

MM: no

MS: no, ok, why?

MM: she said she beliefs that that one, that sickness is for this eh… modern medicines that we have

MS: ok

MM: but if the child is having a sickness that is knew for traditional healer then they will do that

MS: what are those illness?

MM: ok, she said things like seizures, sometimes you know children do have seizures, so those kind of things if you go to this eh… traditional healers they have medicine for them

P011 adds stuff in Mandinka

MM: she said if you, if you… are pregnant, sometime when you go to the marabouts they can eh… make something for you, just like a… juju or safou, then if you tight it on you, then your child will not have those kind of things, these seizures and other things, your child won’t have

MS: ok, ok

MM: she said they are doing all that but god is the one controlling everything

MS: ok, ok, eh… and then, what does she think of the services of the health center.

MM: what she is saying is, you know, ehmm… the saddest thing about the health center is sometimes you leave everything, go with the sick patient there, from morning you’ll be in the queue until afternoon, then they will just tell you there is no medicine left, so you go and buy to the pharmacy, so… and you have other things to do but you, you join the queue since the morning, till late hours they say no medicine, then if you are there without money, how are you going to buy those medicine? Very sad, and also your works, you have chores to do, you have chores to do just like laundry and cooking and other… cooking and other things, you don’t do them, you know? They have work at the garden, you know? watering the garden, all those things you leave them, then you go waste your time, they say no medicine

MS: ok

MM: yeah, that is why she is saying it is very sad

MS: hmm, and the nurses and the doctors are nice with her?

MM: so… what she is saying is that people are not the same, some people have patience and some don’t have patience

MS: ok, ok… then, and then, we are reaching the end of it sorry, can we know if she has ever known someone with acute rheumatic fever?

MM: no

MS: no… and has she ever heard about it?

MM: she said her children no one has ever had it, but sometimes… she saw some people who there children do have it

MS: aha… and how do you know if they have it?

MM without translating: no you know when you have it your mouth is at your mouth it should be something like that is burn by hot water

MS: hmm… burn by hot water?

MM: is like the thing when you put very hot water inside your mouth how your tongue used to be, or that is how it is, because of the fever sometime

MS: ok… and has she ever known someone with rheumatic heart disease?

MM: no

MS: and has she ever heard about it?

MM: yes she heard about it, she used to heard that they said this person is because Rheumatic heart disease killed the person

MS: oh… and was it a young person or old person?

MM: old person

MS: old person, and how does she know that someone has Rheumatic heart disease?

MM: nono she heard that people said that is what killed those people, but she never experienced it on her, or meet anyone with it

MS: so does she know what symptoms does people with rheumatic heart disease have?

MM: she said she never saw any person with that, but she heard that they said this person is dead because of rheumatic heart disease

MS: ok

MM: this person died because…

p011 speaks Mandinka

MM: she said the reason why sometime when she heard that someone is sick she does not go to visit them is because some people when the go visit sick patient what they see they just be going around telling people this is how I find this person in the household, that is what I see he needs to do that

MS: hmm, ok, and my last question is, when she has sore throat, does she, how does she feel, does she feel other things apart from sore throat?

MM: she said because of the lack of eating you know swallowing is difficult, so that may cause her lose weight, those are all

MS: ok, so no fever, no joint pain…

MM: no

MS: ok, so that is it if there is anything else that you would like to say?

MM: she said all she wants to say she said

MS:aha, and do you have any questions?

MM: no

MS: ok then abaraka bakeh for your time this was very good.

# P012/13

MS: now this is recording, abaraka bakeh for participating in the study, ehm… can we start the interview by telling me a little bit about yourself? Like how old are you? How many children do you have?

MM translates (and does so if I don’t say anything)

P012: twenty-five

MM: twenty five

MS: hmm, like me, haha

P012: haha

MM: grade nine, she stopped at grade nine

MS: the school?

MM: hmm, ok, she is not having a child, it is the first pregnancy

MS: ah ok, congratulations

P012: hmm

MS: eh… and eh… is she has experienced sore throat recently? You?

P012: no

MM: him

(points at a little boy in the room)

MS: him?

MM: hmm

MS: eh… and what is your relationship to him?

MM: the younger brother

MS: younger brother

MS: and you are the oldest sister?

P012: no

MS: no, which… how many siblings do you have, in total?

MM: ok, so they are eight,

MS: eight siblings

MM: aha

MS: and he… you are not the oldest… are you the second oldest?

MM: the second

MS: ok, good, and the tribe is…. Mandinka?

MM: Bambara

P013: bambara

MS: Bambara, ok, and language? Bambara aswell?

P013: mali

MS: which one?

P013 bambara

MM: bambara but they cans peak Mandinka (p013 speaks) ok, she said she cannot speak Bambara, she only speak Mandinka because she was born here

MS: ok… but the tribe is bambara

MM: hmmm

MS: ok good, Ehmm… Ok, and then can you tell us eh… like what happened when he had sore throat the last time? When did he have it, eh… how did you know that he had it?

MM: he used to say that he is having sore pain, yes

MS: and when did it happen

MM: more than a month

MS: more than a month ago? Ok, eh… and they knew he had sore throat because of the pain? And what is … like can you ask them how do they define sore throat? What is sore throat?

MM (p013 and p012): ok, so the woman said, what they know about it is, you know this porridge you know porridge?

MS: this?

MM: porridge

MS: porridge yes

MM: when you prepare porridge we use the spoon to drink, then after if you are done, you don’t wash the spoon and you start liking it, like that, it can cause sore throat

MS: hmm

MM (p012 and 13): and she also said if you eat with people in the same bowl and just wash your hand and wipe it on the fence or something then that can also cause sore throat

MS: hmm, ok, ok, ehm… and what did you do when he had sore throat?

MM (p012): she said she going to her garden and bring some herbs, then she boiled it, then after you just cover yourself with blanket, you open your mouth so the flame would go inside then something would just come out, then it goes away

MS: aha, aha, ok, and and which herbs are those?

MM: no she can not tell me the name, I asked but she said it is in the garden but she can not say the name

MS: ok ok, and did the sore throat stop after he take the herbs?

MM (p013): she said sore throat is two types,

MS: hmm

MM: the other one is just the pain of the throat but the other one just like the old man explained, the small… this thing inside, is that one just fall down, then after go and use the spoon put black pepper there, then try to lift it up, so it will go back to initial position. So those are the two types of sore throat.

MS: ok… so one type is only here, and the other type is only looking?

MM: yes

MS: ok, and which one did he have?

MM: the other one, he has the other one, she said that one if you don’t remove it, if you don’t do that, so that that thing would come out, if it goes inside the stomach it can kill someone

MS: ok, ok, ehm… and ehm.. do you think that sore throat is dangerous?

P013 answers with a very high voice

MM013: yes, she says it is dangerous, she said when you have it you can not eat, you can not drink, so it is dangerous, she said sometimes you can’t, you can’t even talk.

MS: you can’t even talk

MM: yes, she said when you have it if are… if you don’t rush to go to get medicine for it you can, you can became died

MS: ok, and ehm… the like with him the medicine was the tea, he didn’t take anything else?

P013: no

MS: no, ok, and has he had other sore throats other times? Before that?

MM013: yes before that

MS: yes? how often does he have it? Normally?

MM013: sometimes, one sometimes in a year

MS: one sometimes in a year, ok, and the other children?

MM013: no

MS: no? only him? Ok, Hmm like, he is the only sibling that has ever had sore throat?

MM: yes

MS: ok, and eh… do you know eh… do you know what other people do when their children have sore throat? In Sukuta?

MM013: she said eh.. when they ask her for help then she would gather and bring the herbs to her, but if they don’t tell her then she does not know

MS: she does not know, ok, and does she know why is he the only one to have sore throat in the family?

MM013: it is just sickness…. She said once his brother once had it, but it was long time, it was a long time ago

MS: ok, and do you know why did he get it the last time?

p013: no

MS: no, ok, ehmm.. and have you ever been to a marabout or a hospital before?

MM013: she is saying how her husband is curing it

MS: with the herb?

MM: hmmm

MS: ok, good, and do you know like, related to what I asked before, what do other people think about getting sore throat? Like before I asked about how do they treat it but…. How do they get it?

MM: but I think they have already answered that

MS: yes? Like they… but they said their opinion, but about other people, yeah

MM: ah ok

Translates

MM: she said sukuta that is what they believe, after you wipe your hands against the wall

MS: hmm

MM012: so that is what she said, you know sometimes some people they would not use both hands to wash their hands, so they only use one, you put water, then you wipe it on to something, put water again, so they say sometimes they would even tell you to stop it because if not someone might get sore throat

MS: aha, so can you ask her like, if I don’t wash my hands, if I do like this, I give you sore throat or I get sore throat?

MM013: she said when you do it, maybe someone might get it, or maybe you might get it

MS: oh… so it is both possibilities? Ok, ok, I understand

MM: hmmm

MS: can we know how far away are you from the hospital?

MM: four or three minutes, you know it is not far

MS: like walking? Ok, and that is the sukuta health center? And the pharmacy?

MM: pharmacy is at the back here

MS: so…

MM: one minute walking

MS: ok, and for marabout or for traditional healer?

MM: they that have one here

MS: you go to this one? Hmmm, well ok, then so when do you go to see the traditional marabout?

MM012: when she has severe headache she go to her father and would give her…

MS: ah so that is your father? Ah ok, and what does he give to you for headache?

MM: they prepare something, you know sometimes they will write it down then after wash it inside water, then that is what he will give her, he had or bad witches

MS: and would they ever decide to go to sukuta health center for other reasons?

MM: yes they would go there for other reasons

MS: for example?

MM: ehm… her pregnancy? Haha for example

MS: ah ok

MM: yes they would go there for antenatal

MS: ah ok, and to the pharmacy?

MM013: pharmacy when they prescribe medicine for them at the hospital they just go to the pharmacy and buy it

MS: ok, and who goes with the children normally? To the hospital or the pharmacy?

MM: aminata

MS: ok, and is that the oldest sister?

MM: her brother’s wife

MS: the brother’s wife?

MM: hmm

MS: ok, and why is she in charge of doing that?

MM012: ok, she said you know they are here together, so some of them their mothers are around, so if they are sick their mother will take them to the hospital,

MS: but this one is the wife of a brother

MM: yes

MS: ok and who pays for the healthcare?

P013: the father

MS: and what do you think about the service in sukuta?

MM: medicine haha… they don’t use to have the drugs

MS: they don’t give them drugs?

MM: no, they don’t have it, they go there they don’t have it so they have to go somewhere else

P013 talks

MM. they will just prescribe it they you have to go to pharmacy to buy them

MS: hmm ok, ok, and then can we ask if they have ever heard about someone having acute rheumatic fever?

MM013: yes she said that once her daughter’s child once had it,

MS: aha, and how… how did she know that she’d had it?

MM: because you know when you have it…

P013 speaks and p012 also (they say palpalta and balokando a lot)

MM: she said the body was hot, until if you put water, you put the daughter inside water, you see something like flame coming out

MS: if I put butter?

MM: nono, take this shirt, put it in water and place it on the body you see flame coming out

MS: ok, ok, and did he had other symptoms?

MM012: and headache too

MS: headache, ok, and what happened, what did they do when that happened?

MM013: they took her to the hospital, you know most of the time they give her the paracetamol, but she said that paracetamol was not helping, they end up giving this herbal treatment

MS: this?

MM: herbal treatment, the local medicine,

MS: aha, from the father? The marabout gave it?

P012: aha

MS: And which treatment was it? Do they know?

MM: no no no,

MS: and that worked?

MM: yeah, she said it worked

MS: how long did it take to work?

MM: less than four days, three days

MS: ok, and that never happened again?

MM: ah ok, it was not long

MS: and when did it happen?

MM: among three weeks

MS: three weeks ago, ok, and now she is fine?

P012: hmmm

MS: ok, good

MM: she said she was a side of the bowl (no entenc 20.54) and she was being sick because of the fever

MS: aha, and have they ever heard about RHD?

MM: no

MS: no? never heard about it never known anyone?

MM: no

MS: ok, so do they want to add anything else?

MM: no, she said only waist pain is the one disturbing everyone here

MS: everyone here? Young people and old people?

MM: hmm, old ones

MS: old ones ok, and do they want to add anything?

MM: no

MS: ok then abaraka bakeh for your time, this is the end of the interview, thank you.

# P014

MS: eh… thank you very much for participating, ehm… can we start by… can you tell me a little bit about yourself? Like, how old are you, how many children do you have?

MM: twenty six, four children

MS: hmm, how old are they?

MM: the oldest is seven,

MS: aha

MM: the one… the second one is four years

MS: four

MM: four yes, the third one is two, the last one is six months

MS: six months wow, and what is your occupation?

MM: she is not working, but sometimes she would go to the market to sell

MS: ok, what does she sell?

MM: hmm?

MS: what does she sell?

MM: this onions

MS: nice, and what education did she have?

MM: she went to school in Mali

MS: in Mali? Ok

MM: she stopped at grade nine

MS: ok, and what is your tribe?

MM: Bambara, is Bambara yes

MS: bambara, ok, and you speak mandinka here?

P014: yes

MS: aha, ok and who had the sore throat most recently? Which child did…?

MM: the one who is in four years

MS: aha, and when did it have it?

MM: is… it take some time now… six months now

MS: six months ago? Ok, and what happened? Can you tell us what happened? When it started to have sore throat, how did you know that it had sore throat?

MM: she said the child said eh… the neck is paining, and whenever they are eating she used to cry, and when she is drinking also she would cry

MS: oh… ok, so did the child complain to her or did she see first?

MM: the child complained that the throat is paining, but after that she also observed whenever she is eating or drinking she would cry

MS: hmmm, for how long did she have it?

MM: three days

MS: three days, and how did it stop?

MM: she went to the hospital and give medicine

MS: hmm, which kind of medicine?

MM: she said she can not remember the medicine because even the prescription part is lost.

MS: ok, and did they give you the medicine at the hospital?

MM: yes

MS: ok, and who went with the child to the hospital?

MM: she went

MS: and who paid for it?

MM: the father

MS: the father ok, ok, and did you try to treat it first at home? Or…?

MM: no

MS: no, ok, and ehmm… how did she think that the child had the sore throat? Got the sore throat?

MM: she can not tell

MS: ok, and the other children also had the sore throat?

MM: no

MS: and before this time, has the boy had sore throat before?

MM: she said before the small… little girl has it, that is the first…

MS: ah it is a girl sorry, ok, sorry

MM: the four years girl, before she had it, the first child have it, and she also once had it but that one was a long time

MS: ok, and what happened when the first child had it?

MM: he said the… for the oldest child, that one did not disturb him, because the night he said the throat is paining, then the next morning he said it is fine, but for her it disturbed her a lot until this old man here prescribed, tell her to go and, you know, take some herbs from the tree then she do what she said, you will just pound it then after put pepper there, then you put it here then after pus will come out, so that is what she did

MS: ok, that is what she did, and why did she not do that with the four year girl?

MM: she said you know, she is small, and this one is hot water, so it is not safe to do that

MS: hmmm, ok, ok, and does she think that the sore throat can be dangerous for the child?

MM: she said it is dangerous because anything, are to eat or drink is painful, so you have to force yourself to eat, and for children maybe they will not be able to do that, if pains they will just leave it out

MS: ok, ok, ehmm… and does she know what other people in the community do when the children have sore throats?

MM: no

MS: no, ok, and… how far away are you from sukuta health center?

MM: it is less than thirty minutes

MS: less than…?

MM: thirty minutes, she said if you go outside but you don’t stop anywhere, is less than thirty minutes to go to the health center

MS: ok and to the hospital?

MM: pharmacy

MS: (they both laugh) ok, the hospital is that aswell and to the pharmacy?

P014: ten fifteen minutes

MS: ok, and for children you would always go to the hospital first right?

MM: yes

MS: ok, and have you ever heard about acute rheumatic fever?

MM: ok she said, ehmm, one of, her child that is two years old once had fever, but she can not tell you that is that but that time she went to the hospital and they give her medicine, the fever still did not go, then after she went to the pharmacy after, then the same thing, then she went back to the hospital again, that is the time the tablet, that is the time the fever goes away

MS: ok, and did they give her something different that time? Does she know what?

MM: no

MS: ok so she does not know why?

MM: no no, she does not know

MS: ok, and the last time that the four year old had the medicine how long did she take the medicine for? When did they stop giving medicine to her?

MM: three days

MS: three days, and that is what they told her in the hospital or when the… did the pills finish, or when the sore throat went away did they stop giving it or…?

MM: they just give her the drugs, but did not tell whether she should finish it or she to stop giving

MS: so they stop giving when the sore throat went away?

MM: yes

MS: ok, so… has she ever heard about Rheumatic heart disease?

MM: no, she had it but she had never seen it

MS: oh, and how do you know you had it?

MM: she said, she just heard it they said there is this heart disease but she has never seen someone who had it or she does not know if they had it, what… how the… the condition is, what they look like or something, just heard that the disease is here

MS: and she does not know how to know if someone has it?

MM: yes

MS: ok… then do you want to add anything to this interview that we have not asked?

MM: no

MS: does she have any questions?

MM: no

MS: then this is abantah, sorry for the delay abaraka bakeh, you can go back to cook

# P015

P015: no problem

MS: abaraka bakeh for speaking with me to let me learn about what you know about sore throat, so can you tell me what you eh… you do when people come with sore throats to you?

MM translates (and does so if I don’t state differently)

MM: he said when people come for sore throat, the complain of sore throat, they do give them medicine, but there are different type of medicine

MS: hmm which type…?

P015 starts speaking without waiting for translation from MM

MM: ok, he said, they would take eh… some tree either a leave or something, then he would recite something on it, then after reciting that thing on it, he would take eh… pepper, with salt and “pound” it until it is powder, then you just put your finger like this and put it on the tongue, then after that he just take hot water and just like you are blowing something, then the pus inside will all come out

While explaining this p015 inhales heavily making a demonstration with the head facing the floor /the hypothetical bucket with hot water/

MS: ahh, and eh… which tree is it?

MM: you can use mango tree,

p015 speaks more

MM: he said there is one tree, also the bambarans call it GRO

MS: gro? aha

MM: yeah gro, so but I don’t know the name in Mandinka, haha I don’t know that tree, they use that tree also

MS: ok, ok

P015 keeps talking

MM: the sore throats have many different ways, sometimes some people have it through witchcraft,

MS: with cough?

MM: hmm, some, you know, is like maybe if you do believe that there are certain wind that when you… when that one touch you it can give you a sickness,

MS: ohhh

MM: yes, so he said some are caused by that

MS: ok

MM: those kind of wind

P015 keeps talking

MM: many people, especially the white, they don’t understand these kind of things

MS: aha, is it the jinn? The wind is it like a jinn?

MM: yes it is the jinn,

MS: aha

MM: these vampire sometime, you know here, Africa here, people do believe that they exist so the vampire also, those are the people also can give you sore throat

MS: hmm, and is there a reason for which the vampires give sore throat to people?

MM: ok, yes, there is a reason for it

(p015 keeps talking)

MM: he said you know there is a reason, you know is Africa, maybe you have something which I been want you to have for I am jealous of you, I can do that to you whilst I have the power, I can give it to you, just to disturb you

MS: and who has the power?

MM: the vampires,

MS the vampires

MM: and some people are there born the same there, they are spiritualist, you know, they can see beyond what we see, those kind of people

MS: and can you protect yourself against the vampire’s witchcraft? For konkonuto dimo?

MM: yes

MS: and how?

MM: he said in Africa here, you have two type, you can write it on a paper and, you know, put it on your waist, or you can put it in water and bath with it, then you will not have it

MS: ah… ok, ok, and like, who comes normally to see you for sore throat?

MM: both adults and children

MS: both adults and children

MM: he said also sore throat also has many types of … it has different types

MS: hmm, ok

MM: there is a sore throat that will make your neck swallow, just swallow like that, but that can, that can be found inside someone’s body

MS: ohh

MM: ok, he said that one, eh.. there is no… nothing like vampire or this jinn, there are no, there are not involved, that one is inside yourself

MS: and where inside you?

MM: he said sometimes you can sit and feel like something is moving inside your body, yes

MS: and that is the sore throat?

MM: he said that can cause sore throat

MS: and is the treatment for children and adults the same?

MM: no, the treatment for children and adults are not the same

MS: hmm, and what is it for children?

MM: he said there are some kids, you know sometimes I’ve met they will have had fever, some will be having stomach ache, some will be having headache, yeah

MS:hmm

MM: or your heart will be beating very fast

MS: and is this related with the sore throat?

MM: nono they are not the same

MS: no, and the children complain about sore throat or have sore throat when they come to see you? Or..?

MM: yes, they do come, (p015 talks) he said for kids when they come, he normally recite something and start just touching the flesh like that, massaging the neck where the paining were

MS: ok, cause like, some people have told me about the Safou that you put around your neck? Does he do that?

MM: people have different ways in treating sore throat,

MS: ok

MM: so that one, you can do that, you can put it on the neck

MS: he likes it?

MM: yes

MS: is good, does he think it works?

MM: yes, yes

MS: ok, and the Safou works to protect you or to cure you?

MM: ah ok, when it is still on your neck you will not have sore throat

MS: ah ok, so it is to protect mostly

MM: it will remove it out, then after protect you

MS: ah ok, and how did you learn, how did you know learn all this knowledge on sore throat?

MM: from the father

MS: that was also a marabout?

MM: yes

MS: aha, Mandinka marabout?

MM: Bambara

MS: ok, and now you are teaching someone?

P015: yes (Mandinka)

MM: he said he is having his son that he is teaching

MS: ok, and can we ask him if sore throat can be dangerous for the people?

MM: he said yes, it is dangerous, because when you have it you can not eat or drink

MS: hmm ok, and can we finally ask him what does he think about how do they treat sore throats in the health center, or in the pharmacy or in other places?

MM’s phone interrupts interview, he picks it up and p015 stops until he hangs up, then continues speaking

MM: he said, you know, there are lot of sickness which you know, sometimes you will go to the marabout, he will not be able to cure it, you go to the hospital or pharmacy, they cure it, sometimes you go to the pharmacy and they can not cure the disease, and you come back to the marabout, he cure it. But for sore throat, they can not say which one is better, for him, you know he is old, since he don’t go to hospital, so they can talk for his one

MS: hmm

MM: he said ehmm… the white people are taking medicine from trees, and they also take them from trees, it is just the way they process it are different

MS: yeah, ok

MM: yes

MS: and… eh… can you tell him that I went to Serrekunda market and I asked the people from the herbs shop what would they give me for sore throat and they gave me bannah powder, what does he think about that.

MM: he said, ehmm… you know banah is also good, it can cure sore throat, but is all the same but before you go for banah, because sometime to have it is difficult, he can do it without struggling to get banah, so that is why he used not to go for that one (p015 adds something) he said there is a girl who once came to him, before he came to him he went to you know there is the clinic here… around here, sukuta here, it is a big clinic, the girl went there and you know, they admitted her for four days, and she paid eight thousand dalasi, but were not able to cure her, then when she came to him, then only two days, the girl was fine

MS: and this was because…

P015: kankonuto dimo

MS: hmm, and what did he give to her?

MM: he give her some herbs

MS: hmm, and apart of vampires is there anything else that causes sore throat?

MM: he said ehmm… you know sore throat, ehmm, you know this vampires and the wind that I said, if that wind enters inside your body it comes through the throat to come out, so sometimes when it is coming out, it will make small disturbing the lymph nodes here swollen and so if you want to eat something or drink something you can not swallow, so he said there is another one, that used to be here, so that one also you also take the leaf of tree then burn it then after pound it that small, then that powder he will recite something on it, then after you put some in water and drink, then the other one you put ehmm… oil on it then and start putting it here and it goes away. So but he said there is also this knowledge that they also have, maybe you can buy an apple, that apple you recite something on it, if I give you to eat, if when you eat, it will just stop here, it will not go down, it will not come out, unless he want to remove it for you, but you can not do anything, you can not, if you don’t vomit it, everything will stop here, nothing will not pass, nothing goes in, nothing goes out, he said they have that one too

MS: ok hmm

MM: he said, he said also he has not had a sore throat or adult sore throat? (confused - not clear min 20.00)

MS: ok, then does he want to add anything that he thinks that I haven’t asked and he wants to teach me?

MM: he said right now he is busy, people are waiting for him, but if you have time you can still come here and he will sit with you

MS: ok

MM: and he will teach you some of the things, if you want you if you go back you can try them and see

MS: aah ok, ok, I will try, maybe I can try to come on Friday

MM: yes he say on Friday he does not use to work, so you can come on Friday

MS: ok then I will try, abaraka bakeh for your time

P015: abaraka bakeh, salam

# P016

Part 1 of the interview

MS: if you can tell me a little bit about yourself, like how old are you? For example

P016: 34

MS: 34, and how many children do you have?

P016: five

MS: five children, and how old are they?

P016: eh… the first one have eleven years, second one nine years

MS: aha

P016: and third one six years, and the fourth one two years

MS: ok

P016: the fifth one seven months

MS: ah ok, this is the fifth one?

P016: yes

MS: ah ok (both laugh) ehmm.. and what do you work on?

P016: at this moment I work at the salon

MS: at the salon, ok,

MM: hairdresser

MS: aha, nice, and ehm.. how many years of education did you have?

P016: yeah, nine years

MS: nine years, that is until… grade

MM: grade nine

MS: grade nine, ok

P016: yes

MS: ehm… and what’s the… what tribe do you belong to?

P016: Mandinka

MS: Mandinka, and what languages do you speak?

P016: three

MS: three, ok, serer, Mandinka and English?

P016: yes, wolof

MS: wolof, wow, ok nice, ehmm.. what else? And how many other like… are there other parents from this household that live here as well?

P016: yes, they live here

MS: aha, is it different wifes or different families?

P016: no, one family

MS: ok, and how many people live in the house in total?

P016: ehmm… (counts) 18

MS: 18, ok, so… this one had the sore throat most recently?

P016: yes

MS: How long ago did he had it?

P016: maybe three years

MS: three years ago, ok, and what happened? Eh… how did you know he had sore throat?

P016: he used to complain, when the pain started he cried

MS: oh…

P016: yes

Husband speaks to her in Mandinka and she asks me to stop the recoring

Part 2 of the interview

MS: so he started crying before complaining or after complaining?

P016: before complaining

MS: before, so you knew something was happening

P016: yes

MS: and then… did you ask him what is happening? Or…?

P016: yes, I asked him what is going on… he said I feel pain here

MS: aha

P016: when I want to eat something or I want to drink water or… I feel pain here

MS: hmm, ok, and did he feel something else?

P016: of course sometimes his body… a bit hot

MS: ok, hmm, and how did you know that the body got hot?

P016: when I touch his body like here

MS: ok, ok, and what did you do after?

P016: I take this… black pepper, you know our local way, I take hot water and put it in, I leave until when it warm, another (something unintelligible around min 1.00 because the children are speaking very close to the Dictaphone) green tea, when he drink it maybe five or ten or fifteen minutes he will start to say that now the pain is… I don’t feel the pain now

MS: hmm, nice, and do you have to drink or just keep it in the mouth?

P016: no, he has to drink it

MS: drink it, ok… one time?

P016: no, sometimes one sometimes twice

MS: ok, hmm, and then how long did it take for the sore throat to go away? After… that?

P016: maybe sometimes when the pain stop maybe it will have four or five months before another one

MS: ok,

P016: yeah

MS: ok

P016: but… maybe in a year, I can say… maybe he will have it two or three times, or sometimes more than that

MS: hmmm, and this year, he has had it?

P016: of course

MS: two or three times aswell?

P016: hmm… maybe if I can recall, maybe two or three times, if I can recall

MS: ok, and all the times you have used black pepper with water?

P016: yes

MS: and it has worked?

P016: hmm

MS: ok, so after the black pepper and water it stops?

P016: yes

MS: and then… you don’t do anything else?

P016: no

MS: ok, ok and ho… why do you think he had sore throat?

P016: because sometimes when he open his mouth I see something here, but I don’t know what it is because I can not touch it

MS: ok

P016: yeah, but I, I see something, but when he drink that water, that thing disappear, we don’t see it, so I do not know whether the thing used to go inside the stomach, I don’t know, but…

MS: ok, yeah

P016: …yes,

MS: aha, and how do you think he gets the sore throat?

P016: I don’t know that

MS: and do you know what eh… other people in Sukuta think about how you get sore throat?

P016: well something that eh… when you eat family you eat one bowl, when somebody want to wash his hand then you wash it, after washing it, he, you know, here, here in gambia many people when we are eating we use to put water inside the bucket

MS: yes

P016: after eating it, you wash your hand there, so that was, when you wash you make your hand like this, for example this is eh… how it called? This is the eh…

MM: the top

P016: the top! When you wash your hand here after that you make your hand like this

MS: aha in the cup?

P016: yes in the cup

MM: nono, on the sides of the bucket, the top part,

P016: yes the top

MM: they just wipe it like that

P016: so after that, the people you eat with them, so that is our belief, some will say that when you do that some will have this thing

MS: another person?

P016: yes

MS: not you?

P016: no, not you

MS: another one

P016: yes, another one will have it

MS: ok, and do you think that happens? Is this your belief aswell?

P016: yeah yeah yes, we believe that

MS: hmm, and do you know what other people in Sukuta do to cure sore throat?

P016: yes in Sukuta I can say when… if a tall stand (not understand min 4.50) I know seven person all of them use to do this, hot water and black pepper

MS: ah… ok, ok, and do other children here also get sore throat?

P016: yes, sometimes they use to have it

MS: aha

P016: you know… even me I use to have it

MS: ok, and do they have it at the same time as him?

P016: no… sometimes it will not be at the same time

MS: ok, and is he the one that had the most sore throats?

P016: yes, yes

MS: ok, and the other ones have it repeat? Sore throats as well?

P016: yes yes

MS: ok,

P016: they use to have it time to time

MS: time to time, more like… a couple… two times a year…? Or once a year? Or less often?

P016: no… I can say once in a year… or two, but it will not be more than two, if I can recall

MS: ok, ok, aha, and with him is sometimes more than two?

P016: yes,

MS: ok, ok… and does he always complain to you? When he has sore throat?

P016: yes he use to complain

MS: ok.. and you are the one preparing the hot water

P016: yes

MS: ok… and can I know how far away do you live from the nearest hospital?

P016: hm…

MS: like walking for example

P016: yes but I can not say how much… but it is far

MS: ok, hmm, it is far, how much is far? If it is forty minutes walking is it far already? Or…?

P016: yes I can say forty minutes or… or maybe thirty minutes if you don’t stop anywhere, if you just… go direct

MS: aha, ok, that’s far, and the pharmacy?

P016: yeah, pharmacy also is far

MS: ok, how much? Thirty minutes walking? Or..?

P016: no… maybe thirty or forty…

MS: ok, so that’s far

P016: yes, forty minutes yes

MS: and… do you have any marabout or traditional healer?

P016: no I don’t know

MS: you don’t know any?

P016: no…

MS: ok, and the health center? Is..?

P016: health center, hmm…

MS: is it the same as a hospital?

P016: yes

MS: and when you say hospital is it the sukuta health cent…

P016: yeah

MS: ok, I get it, and when the children have had sore throat before, have you taken them to a pharmacy, or to the hospital, or a marabout or a grandma?

P016: no, I don’t think I take them to anywhere, because we have this belief and we use to do it when… when you have it… he said I am feeling the pain here so.. we just go and buy pepper if I told you they have it

MS: yes, ok, aha, and it works?

P016: yes, and it works

MS: and it works, and… are you worried when they have sore throat?

P016: of course

MS: why are you worried?

P016: because the kid use to cry and is very hot

MS: hmm

P016: so… he can not eat, he can not drink, not do anything, just lie down, crying… so I get worried

MS: hmm, and do you think that the… the disease can be dangerous for the children?

P016: yes, maybe it can be dangerous, because someday when they have it they use to have this thing…

P016 speaks Mandinka

MM: they use to have the small focus (not able to understand word min 8.44)

MS: the small what?

MM: the small focus like that

MS: eh… in the arm?

MM: yeah

P016: yeah, they had it here

MM: ah ok on the throat

MS: but.. how is it?

MM: just like in the throat the lymph node will be swollen

MS: so… one one? or more?

MM: no, just these two

P016: yes

MS: ah, ok, aha

P016: so that is why we used to get worry, because when the pain start you don’t know where it is going to end

MS: yes

P016: so what we believe we used to do that, but we don’t know whether that one will work or not, but we do that and that one work, but still, the problem can not solve, because when the pain stop maybe we see it three four month, then we see it again (baby screams very loudly min 9.32) so... that is we use that… that black pepper and water, that will stop it but not… is going to cure it

MS: ok, so… is it always inside you? Or… if it is not cured, what happens?

P016: yeah that is what I explained, maybe you can have this thing here, I don’t know how to cure it, maybe like this

MS: yes

P016: some use to have it here, but before having it you will have sore throat first time, after when the things change

MS: ok, ok, nice yes, ehm… and do you go to the health center or hospital for other diseases? Like malaria or other?

P016: yes we use to go

MS: ok, and have you ever heard about… acute Rheumatic fever? And maybe this is a bit of a medical term so can you translate MM?

P016: no

MS: no… have you ever known someone that has it?

P016: no this one no

MS: ok… and about rheumatic heart disease?

P016 speaks Mandinka

MM: she said none of her kids one has it but she knows someone who had it but that one passed away

MS: oh… a little one or old?

MM: little

MS: how old? You don’t know?

P016: I think around six months, small

MS: ok, and how do you know that the baby had it?

P016: they went to the hospital, and they check the baby, they make a scan, they said baby is having ehm… heart failure

MS: ok, and what else did you hear about this?

P016: I heard that the baby has passed away

MS: ok, but nothing about why did he have it or… you don’t know?

P016: no I don’t know that but I hear them say the baby come with the problem, the time he was delivered

MS: aha

P016: ok

MS: ok, then I think I have asked all my questions… and can I ask where do you buy the black pepper from? Do you buy it from the market or someone… someone special? Or..?

P016: yeah I used to buy it at the market, but we finish and my heart had this problem having salt and …

MS: so you can find it everywhere?

P016: yes

The girl starts grunting very loudly

P016: stop it
